# Recent Advances on Metal-Based Near-Infrared and Infrared Emitting OLEDs

**DOI:** 10.3390/molecules24071412

**Published:** 2019-04-10

**Authors:** Malika Ibrahim-Ouali, Frédéric Dumur

**Affiliations:** 1 Aix Marseille Univ, CNRS, Centrale Marseille, iSm2, F-13397 Marseille, France; malika.ibrahim@univ-amu.fr; 2 Aix Marseille Univ, CNRS, ICR, UMR 7273, F-13397 Marseille, France

**Keywords:** OLED, iridium, platinum, lanthanide, osmium, phthalocyanine, near-infrared emission, metal complexes

## Abstract

During the past decades, the development of emissive materials for organic light-emitting diodes (OLEDs) in infrared region has focused the interest of numerous research groups as these devices can find interest in applications ranging from optical communication to defense. To date, metal complexes have been most widely studied to elaborate near-infrared (NIR) emitters due to their low energy emissive triplet states and their facile access. In this review, an overview of the different metal complexes used in OLEDs and enabling to get an infrared emission is provided.

## 1. Introduction

During the past decades, a great deal of efforts has been devoted to improving the device-stacking as well as the materials used to fabricate organic light-emitting diodes (OLEDs). This extensive work is notably supported by the promising prospects and the wide range of applications in which OLEDs are involved, ranging from lighting to flat panel display and signage technology. These intense research efforts are also supported by the fact that OLEDs have been identified as the next generation of devices that could replace the present inorganic technology developed on glass substrates, heavier than plastic substrates and shatter-prone [1,2]. As the main advantages of OLEDs, these devices can be lightweight, designed on flexible substrates, and extremely thin. Since the pioneer works of Tang and Van Slyke in 1987 [3], a clear evolution of the materials used to fabricate OLEDs has been observed, and the light-emitting materials have not been exempted. In this field, three main periods can be identified, corresponding to the development of the first generation of light-emitting materials (fluorescent materials) rapidly substituted by the triplet emitters (phosphorescent materials). In 2012, a breakthrough was achieved by Chihaya Adachi who evidenced the benefits of the third generation of emitters, that is, the Thermally Activated Delayed Fluorescence (TADF) emitters which could easily compete with the metal-based phosphorescent light-emitting materials while being metal-free [4]. This evolution of structure is the result of the observation that the elongation of the excited state lifetime of emitters and the possibility to harvest both singlet and triplet excitons with phosphorescent and TADF materials can greatly improve the overall electroluminescence efficiencies of electroluminescent devices, an internal quantum efficiency (IQE) close to the unit being achievable. More precisely, for fluorescent materials, only the singlet excitons can contribute to light emission, limiting the IQE to 25% [5]. Considering that the singlet and triplet excitons are produced in a 1:3 ratio according to spin statistics, and that 75% of the generated excitons are lost in non-radiative processes with fluorescent materials, rapidly transition metal complexes in which both singlet and triplet excitons can be utilized for light emission have rapidly discarded the first generation of emitters [6]. By an efficient intersystem crossing, all singlet excitons can be converted to triplet excitons, optimizing the IQE to 100%. Indeed, by the strong spin-orbit coupling, existing within transition metal complexes, a change in spin state can occur, and transitions that are formally forbidden in non-relativistic quantum theory can take place [7]. However, if this strategy is appealing, the efficiency of the intersystem crossing is dependent on the presence of a transition metal inside the emitters, giving rise to numerous critical issues. Metal complexes displaying a phosphorescence process at room temperature are typically based on iridium, platinum, and osmium, and the scarcity and the high cost of these metal precursors are on the basis of numerous questions concerning the viability of this strategy. From a synthetic viewpoint, it has to be noticed that neutral *tris*-cyclometalated iridium complexes can only be synthesized with reaction yields ranging from 15 to 25%, drastically elevating the final cost of the emitters [8,9,10]. To address this issue, *bis*-cyclometalated iridium complexes have been proposed as alternatives as they can be synthesized in high yields. As a drawback, the use of an ancillary ligand considerably reduces the stability of the resulting complexes, the binding of the metal center to the ancillary ligand being much weaker than that of the cyclometalated ligands [11]. As these complexes only comprise of two C-Ir bonds, their stability is adversely affected and the device lifetime of the corresponding OLEDs is reduced. As another alternative that has been explored, ionic iridium complexes can be synthesized in quantitative yields, and face to this consideration, the use of soft salts composed of cationic and anionic iridium both contributing to light emission has been examined by Thompson and coworkers [12,13,14]. However, the drawback of this strategy is the low tunability of the emission wavelength, the latter being imposed by the complex displaying the smallest bandgap. Face to the high cost of transition metal complexes, alternatives have been researched, and zinc [15] or copper [16] complexes have been proposed as emitters. Besides, performances of zinc complexes are far behind that obtained with iridium complexes due to their fluorescent nature and the impossibility to design neutral copper complexes constituting a severe limitation by the presence of a mobile counterion inside the emissive layer. In 2012, Chihaya Adachi reported purely organic molecules displaying excited state lifetimes comparable to that obtained with transition metals while being metal free. To get this result, purely organic molecules have been designed so that the energy gap between the triplet and singlet excited state (S_1_-T_1_) is small; due to this the electron from the triplet state can be thermally upconverted to the singlet state by reverse intersystem crossing so that an internal quantum efficiency of 100% can be obtained [17]. Contrarily to the phosphorescent materials in which the radiative decay occurs from the triplet state, deexcitation of the TADF materials occurs from the singlet state, giving rise to a fluorescence process.

Parallel to the selection of the light-emitting materials that are used to produce light, control of the emission color is a second parameter to consider, and OLEDs capable of emitting light in the deep red/near-infrared (NIR) region are still scarce. In this field and due to the presence of numerous orbitals, transition metal complexes have been extremely popular to produce a deep red/NIR emission [18,19,20,21,22,23,24]. Indeed, metal complexes can emit in the near-infrared region, thanks to their low energy emissive triplet states. Besides, due to their low energy gaps, device performances remain limited, and platinum complexes have been the most widely studied complexes for producing a deep red/NIR emission [25]. However, their square planar structures, their long-living excited-state lifetimes favorable to triplet-triplet annihilation have adversely impacted their practical use in OLEDs [26]. Parallel to metal complexes, purely organic emitters have also been examined, addressing the toxicity and cost issues [27]. However, it has to be noticed that the availability of organic materials emitting beyond 700 nm is far behind that of organic materials capable of absorbing beyond 700 nm. Advances in NIR emission is however highly researched, based on the numerous emerging applications requiring an emission at these specific wavelengths, such as the communication networks [28,29], night-vision devices [30], sensors [31], and more generally all the military systems used for defense (detection, surveillance, and tracking of targets). Concerning non-military applications, portable thermal imaging camera [32], bio-imaging [33], thermal phototherapy [34], and recently photopolymerization [35,36,37] in the NIR region can be mentioned as the main applications. In fact, development of highly emissive NIR OLEDs is hampered by the “energy gap law”, which specifies that due to vibrational couplings and vibrational overlaps existing between the S_1_/T_1_ states and the ground state S_0_, non-radiative deactivation pathways can take place, and these processes are reinforced for molecules possessing small energy gaps [25,38]. Therefore, NIR emitting materials are perfect candidates giving rise to numerous intrinsic quenching mechanisms. Considering that the development of NIR emitters is a rapidly evolving research field and that strong demand for highly emissive NIR OLEDs exists, in this review, an overview of the different metal-based OLEDs reported at present in the literature is presented. The different strategies developed to improve the performance of a device are also detailed. It has to be noticed that according to the Commission Internationale de l’Eclairage (CIE), the NIR emission extends from 700 to 1400 nm, the first value (700 nm) corresponding to the end of the response of the human eye [39].

## 2. Metal Complexes Using NIR Emitters

As mentioned in the introduction section, platinum complexes have historically been among the first metal complexes to be explored for the design of visible light OLEDs. Logically, works were also directed towards the design of NIR emitters with this metal.

### 2.1. Platinum Complexes

**Figure 1:**
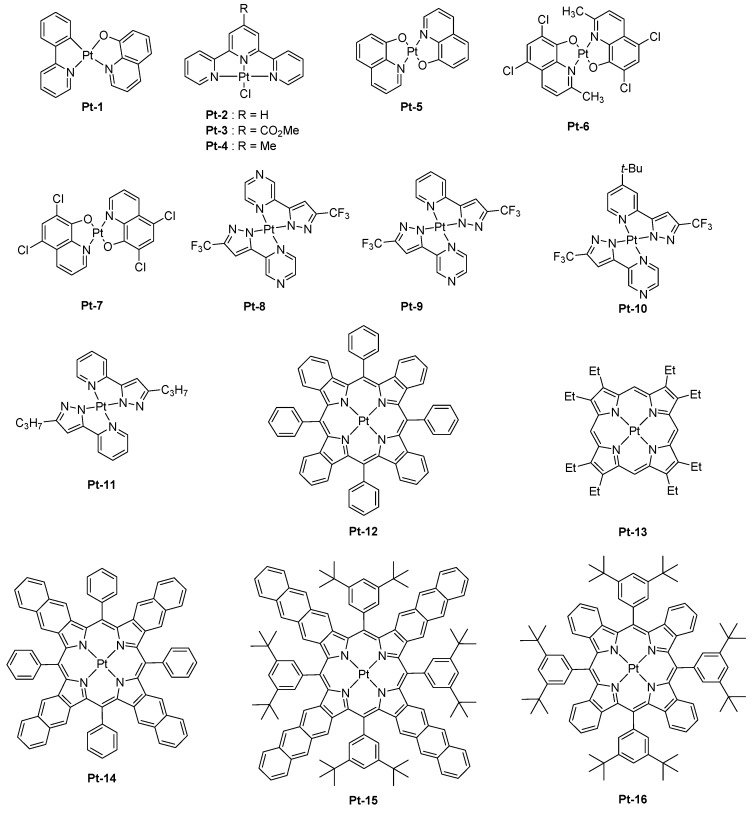
Pt-based near-infrared (NIR) emitters for light-emitting diodes.

Capitalizing on the remarkable performance of *tris*(8-hydroxyquinolinato)aluminum (Alq_3_) reported by Tang and VanSlyke in 1987 [3], a platinum complex **Pt-1** comprising an 8-hydroxyquinoline ligand was proposed in 1995 as a NIR emitter (see Figure 1) [40]. Interestingly, the photoluminescence quantum yield (PLQY) in the solid state was low (0.3% compared to 10% for Alq_3_). Besides, OLEDs were fabricated with this material, and different device structures were examined. Best performances were obtained by using the following device structure: indium-tin-oxide (ITO)/*N*,*N*′-diphenyl-*N*,*N*′-bis(1-naphthyl)-1,1′-biphenyl-4,4″-diamine (NPB) (40 nm)/4,4’-Bis(carbazol-9-yl)biphenyl (CBP): **Pt-1** (10 wt %, 40 nm)/bathocuproine (BCP) (40 nm)/Alq_3_ (40 nm)/Mg:Ag (1:10, 100 nm), and a peak power efficiency of 0.16 lm/W was determined. Higher external quantum efficiencies were obtained while using terdentate ligands for the synthesis of platinum complexes [41]. Contrarily to **Pt-1**, **Pt-2-Pt-4** are square planar complexes, and this specific geometry is favorable to the formation of triplet excimers [42]. This is notably demonstrated by comparing the photoluminescence (PL) spectra in solution and in thin films for the three complexes. A red-shift of about 40 nm was evidenced for all complexes, consistent with emission from the excimer. In a conventional device structure consisting of ITO/*N,N*′-bis(3-methylphenyl)-*N,N*′-diphenylbenzidine (TPD) (70 nm)/CBP (20 nm)/emissive layer (EML) (60 nm)/2,5diphenyl-1,3,4-oxadiazole (OXA) (30 nm)/Ca, an emission peaking at 720, 715, and 705 nm could be determined for **Pt-2-Pt-4**-based devices, respectively. For **Pt-2**, the emission detected beyond 750 nm represented 40% of the total electroluminescence (EL) emission, and a tail extending until 900 nm could be detected. Following this initial work, the same authors revisited **Pt-2** in a new device structure (ITO/75 wt % TPD: 25 wt % PC (60 nm)/CBP (10 nm)/**Pt-2** (30 nm)/OXA (30 nm)/Ca/PbO_2_) and examined the influence of the cathode as well as the electron injection layer (EIL) on the performance of EL [24]. Best OLEDs were fabricated while using Ca as the cathode and PbO_2_ as the electron injection layer, and an external quantum efficiency (EQE) of 14.5% was obtained. In this early work, authors demonstrated that the insertion of a thin buffer layer of PbO_2_ as the EIL could not only minimize the mismatch between the cathode and the electron-transport layer (ETL) but also could reduce the width of the recombination zone by facilitating the electron transportation until the CBP/**Pt-2** interface. Benefiting from the two effects, the driving voltage could be drastically reduced. A broad EL emission extending from 620 to 760 nm with a full width at half maximum of 140 nm was determined, and the portion of NIR emission represented 50% of the total emission. From an emission viewpoint, a similar result could be obtained while designing *bis*(8-hydroxyquinolato)platinum(II) derivatives [43]. Upon increasing the dopant concentration from 1.5 wt % to 10 wt % for **Pt-5**, **Pt-6**, and **Pt-7**, a red-shift of the EL emission was found, resulting from the formation of excimers at high complex concentration. As observed with **Pt-2**, the three complexes produced a NIR EL emission with the main peak centered between 650 and 702 nm, together with a shoulder in the 720–755 nm region. One of the key-element to fabricate highly emissive OLEDs is the photoluminescence quantum yield (PLQY) of the light-emitting materials, and this characteristic is difficult to get for materials emitting in the NIR region. As mentioned in the introduction section, the vibrational overlap between the low-lying excited state and the ground state favor quenching processes for NIR materials [44]. This problem could be overcome with a series of square-planar 2-pyrazinyl pyrazolate Pt(II) complexes **Pt-8-Pt-11** which could also furnish highly emissive thin films by the specific horizontal orientation of the molecules [21]. By controlling the π–π stacking interaction between complexes in the solid state, an emission at long wavelength could be achieved, and a peak EQE of 24% could be realized with **Pt-8**. As specificity, the four complexes investigated in this study were not emissive in aerated and deaerated solutions at room temperature but highly emissive in thin films. PLQYs determined for **Pt-8**, **Pt-9,** and **Pt-10** were high and were 81, 55, and 82%, respectively. Examination of the solid-state packings of complexes **Pt-8-Pt-10** and **Pt-11** revealed an ordered arrangement of the molecules in thin films, resulting in an organized transition dipole distribution. By performing an angle-dependent luminescence measurement, a preferred horizontal orientation of the transition dipole could be evidenced for all materials. By theoretical calculations and due to the aggregation in the solid state, the close packing of complexes gives rise to the formation of dimers, trimers, etc. In these aggregated structures, it could be determined by theoretical calculations that the HOMO level was dominated by a dz_2_ contribution whereas the LUMO level is mainly centered on the ligand π* orbitals. By the specific orientation of the complexes in thin films and the formation of infinite aggregated structures, the limitation imposed by the energy gap law could be overcome.

In this series, the most representative example is the complex **Pt-8** that could produce an EL emission at 740 nm with an EQE of 24%. Seventy-eight percent of the EL emission was located beyond 700 nm. This percentage decreased to 42 and 33% for complexes **Pt-9** and **Pt-10**, respectively (See Table 1). The last class of platinum complexes being examined to produce a NIR emission is the porphyrins and more precisely the benzoporphyrins. Numerous Pt-based porphyrins exhibiting an emission located in the 630–650 nm region have been reported in the literature [6,45,46,47,48,49,50,51,52,53]. To drastically red-shift the emission of porphyrins, the introduction of benzopyrrole moieties in the porphyrin scaffold is required, providing tetrabenzoporphyrins. Owing to a more extended π-conjugation of the porphyrin core and the introduction of bulky groups to the *meso*-positions of the porphyrin core [54], a significant red-shift of both the absorption and the emission spectra could be obtained. Compared to the previous Pt(II) complexes reported in this review, the rate of the intersystem crossing between the singlet and the triplet states, as well as the rate of the radiative decay from the T_1_ state, were increased in metalloporphyrins, limiting the adverse excited state quenching processes. The first example of Pt–tetrabenzoporphyrin used as a dopant for OLEDs and producing an EL emission in the NIR region was reported in 2007 by Thompson and coworkers [55]. Examination of the photophysical properties of **Pt-12** revealed an emission centered at 765 nm, with a radiative decay rate of 1.3 × 10^4^ s^−1^ and a PLQY of 0.8. An excited state lifetime of 53 μs was also determined. While using **Pt-12** as a dopant for Alq_3_, OLEDs exhibiting a maximum EQE of 3% was obtained, with an EL emission close to the PL emission (769 nm vs. 765 nm, respectively). Device stability of OLEDs was also examined and after 1000 h, OLEDs could retain 90% of the initial luminance while being driven at 40 mA/cm². These results are consistent with the device lifetime determined for another platinum complex, that is, **Pt-13** for which a device lifetime of 100,000 h could be obtained while driving OLEDs at low luminance [56]. In a subsequent study, the same authors optimized the EL performance by introducing a hole blocking layer of bathocuproine (BCP) and by reducing the dopant concentration [57]. Precisely, the dopant concentration could be decreased from 6 to 4 wt %, reducing the concentration quenching and triplet-triplet (T-T) annihilation which is the dominant non-radiative deexcitation channel [58]. Benefiting from these two improvements, a maximum EQE of 8.5% was obtained owing to better confinement of excitons within the emissive layer. However, a marked efficiency roll-off, that is, a decrease of the EQE with the current density was evidenced. By co-doping the emissive layer with an iridium complex, authors could elucidate the mechanism of the efficiency roll-off, which is dominated by the T-T annihilation. This demonstration was carried out by co-doping the EML with the triplet emitter *tris*(2-phenylpyridine)iridium Ir(ppy)_3_ of shorter excited state lifetime than that of the Pt-complex. Indeed, by introducing an efficient cascade energy transfer between the host matrix and the Ir dopant, and subsequently on the Pt-complex, the concentration of triplets on the Pt-complex could be significantly reduced, and a comparison of the EL characteristics established with and without Ir dopant evidenced a severe reduction of the maximum EQE in the absence of Ir dopant, demonstrating thus the reduction of the self-quenching effects. **Pt-12** was also examined in the context of solution-processed OLEDs, and devices were fabricated by using poly(*N*-vinylcarbazole) (PVK) as the host polymer [59]. Carbazole-based polymers are extensively used for the design of solution-processed OLEDs due to their exceptional film-forming and charge-transport abilities [60,61,62]. The minimum dopant concentration to get a NIR emission was 1 wt %, and the maximum luminance of 0.2 mW/cm^2^ was obtained. Performance remained limited due to the simplicity of the OLED architecture, which is a single-layered polymer LED (PLED): ITO/PEDOT:PSS/PVK:OXD-7: **Pt-12**/CsF/Al/Ag. A few years later, **Pt-12** was revisited in the context of a series of nine metalloporphyrins in an effort to understand the effects of both the substituents and the π-extended conjugation [63]. It has to be noticed that the pioneering work of Thompson and co-workers [55] on Pt-tetrabenzoporphyrin have demonstrated the feasibility to elaborate high emissive complexes with this ligand while getting an emission centered around 770 nm, and this initial work paved the way for additional studies devoted to extending the π-conjugation of the porphyrin core and the emission in the NIR region. In this study, all the emitters were used for the design of PLEDs and vacuum processed OLEDs. Several trends could be deduced. First, and as predicted by the energy gap law, red-shift of the emission of metalloporphyrins was accompanied by a reduction of the PLQYs, as well as of the excited state lifetimes [64,65,66,67]. To illustrate this, the emission maximum of **Pt-12**, **Pt-14**, and **Pt-15** shifted from 773, 891, to 1022 nm, with triplet lifetimes reducing from 29.9 to 12.7 and 3.2 μs, respectively [67]. Fabrication of PLEDs with these three emitters furnished devices with EL emissions that coincide their PL emissions, except for **Pt-15,** which was determined to be prone to degrade and for which a contribution in the visible range was detected. As anticipated, EQEs decreased from 2.07, 0.75, and 0.12% for **Pt-12**, **Pt-14,** and **Pt-15,** respectively, consistent with a red-shift of their PL/EL emissions. It has to be mentioned that a low dopant concentration was used, minimizing the aggregation and reducing the concentration quenching. When tested in vacuum-processed OLEDs (ITO/NPB (40 nm)/emissive layer/BPhen (80 nm)/LiF (1 nm)/Al), a significant enhancement of the EL characteristics was obtained for **Pt-12** and **Pt-13**, with an EQE peaking at 8.0 and 3.8%. These results are consistent with previous results reported in the literature [68].

It has to be noticed that no evaporated OLEDs were fabricated with **Pt-15**, this material being not stable enough. Impact of the extension of the π-conjugation on the photophysical properties of porphyrins was also examined, and a series of six porphyrins were designed for this purpose.

**Figure 2:**
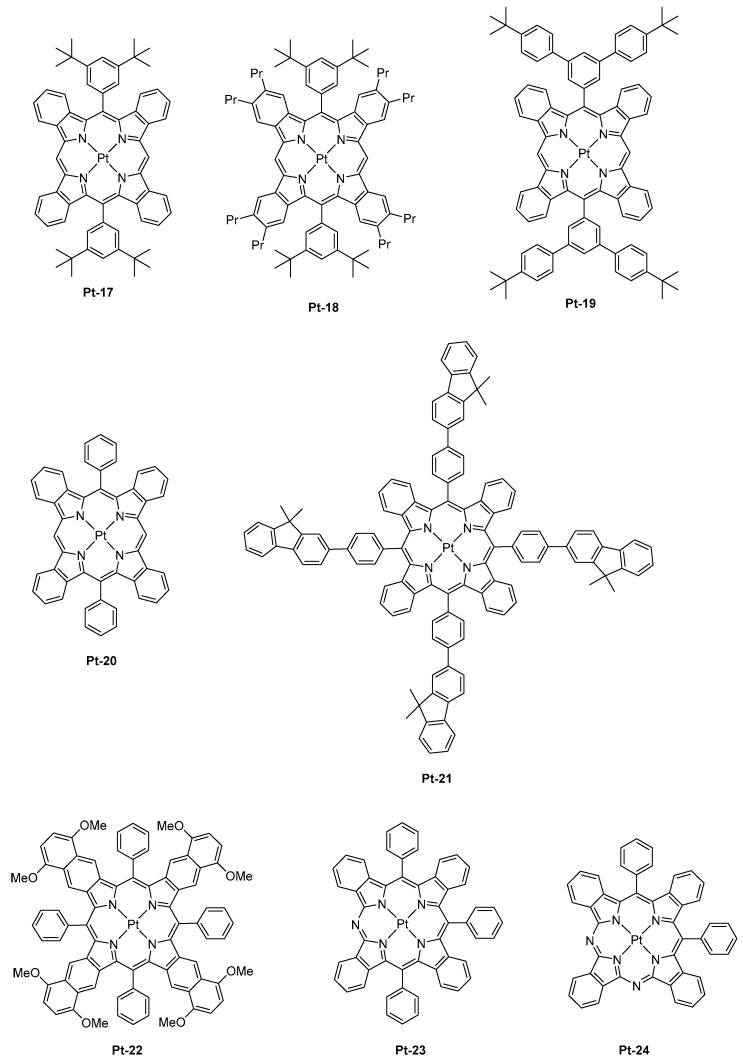
Pt-based near-infrared (NIR) emitters for light-emitting diodes.

First, the comparison between di and tetra-substituted porphyrins revealed both the PLQYs and the excited state lifetimes of tetra-substituted porphyrins to be lower than that of di-substituted porphyrins in solution, resulting from larger degrees of out-of-plane distortion for the tetra-substituted porphyrins. Notably, the PLQY and the excited state lifetime of **Pt-16** (0.33, 32 μs) was lower than that of its di-substituted **Pt-17** counterpart (0.59, 53 μs) or the analogs **Pt-18** (0.45, 52 μs), **Pt-19** (0.44, 52 μs), or **Pt-20** (0.3, 28 μs) (See Figure 2). Similarly, a low PLQY and a short excited-state lifetime were determined for **Pt-21** (0.26, 20 μs) despite the presence of four fluorenes units that are well-known to be highly emissive groups.

While examining the same properties in thin films, a significant elongation of the excited state lifetime, from 50 to 140%, was determined for the tetra-substituted porphyrins. This modification of the excited state lifetimes was assigned to the suppression of non-radiative decay channels in the solid state. By contrast, only minor variations of the excited state lifetime were determined for the di-substituted porphyrins. Therefore, it can be concluded that the photophysical properties determined in solution do not follow the trend observed in thin films and that the examination of these properties in thin films is compulsory. Three notable trends could be determined from the fabrication of PLEDs: (1) the introduction of bulky substituents could increase EQEs by decreasing the aggregation in the solid state. An optimum was found for the substitution of the porphyrin core with *tert*-butyl groups, and an attempt to further increase the size of the peripheral groups did not significantly impact the EL performances. (2) di-substituted porphyrins could furnish higher EL characteristics than the tetra-substituted ones. (3) lifetimes determined in thin films show a good correlation with the PLED efficiencies. In contrast, other trends could be determined for vacuum processed OLEDs: (1) di-substituted porphyrins could furnish lower EL characteristics than the tetra-substituted ones in OLEDs, this opposite trend was assigned to interactions that are different from that observed in PLEDs, especially with the host matrix (Alq_3_ vs. PVK:PBD blend). (2) porphyrins substituted with bulky groups gave lower EL characteristics than the non-substituted ones, and this counter-performance was once again assigned to unexpected interactions with the host matrix. From these results, it was concluded that the non-substituted porphyrins are sufficiently dispersed within the emissive layer (EML) to avoid concentration quenching and T-T annihilation. Among all the emitters tested in PLEDs and OLEDs, the most red-shifted EL emission was evidenced for PLEDs fabricated with **Pt-15**, with emission peaking at 1005 nm and a maximum EQE of 0.12%. In a parallel study, the same authors developed a comprehensive study concerning the influence of the π-conjugation on the position of the EL emission with another set of Pt-porphyrins varying by the number of aromatic rings fused to the pyrrole unit [67]. The conclusions were the same as of the previous ones. By replacing the porphyrin core by a tetraarylbenzoporphyrin (**Pt-12**), a tetraarylnaphthoporphyrin (**Pt-14**), and then a tetraarylanthroporphyrin (**Pt-15**), a red-shift of the PL emission accompanied by a decrease of the PLQYs and the phosphorescence lifetimes was demonstrated, consistent with the energy gap law. Thus, if a PL emission at 773 nm was determined for the parent **Pt-12** benzoporphyrin, a phosphorescence emission clearly in the NIR was detected for the three others, shifting from 891 to 883 and 1022 nm for **Pt-14**, **Pt-22,** and **Pt-15,** respectively. Examination of the EL performance of **Pt-12** in solution-processed OLEDs evidenced devices to exhibit an interesting emission at 896 nm but combined with an extremely low EQE, peaking at 0.4% [68]. Further, the fabrication of multilayered OLEDs with this material showed a maximum EQE of 3.8% at 0.1 mA/cm² and a maximum luminance of 1.8 mW/cm². As a drawback, vacuum-deposited OLEDs showed a severe efficiency roll-off, still resulting from T-T annihilation at high current density. The last examples of Pt-porphyrins used as emitters for OLEDs are the azatetrabenzoporphyrins [69]. Only one article has reported the use of such emitters in the literature, and this is justified by the difficulty of synthesis of such porphyrin derivatives. As a starting point of this study, authors did observe the previous strategy, that is, the introduction of the fused aromatic ring onto the pyrrole unit was an efficient strategy to red-shift the emission, except that the molecular weight of the final compound was too high and the thermal stability too low to be sublimable.

This strategy was also ineffective to shift the emission of tetrabenzoporphyrins centered between 770 nm and 1000 nm. Another possible route to tune the color emission was thus envisioned by Li and co-workers, consisting of the replacement of meso carbon atoms of tetrabenzoporphyrins by nitrogens. Using this strategy, a red-shift emission of the PL of approximately 72 nm for **Pt-23** (λ_em_ = 842 nm) compared to the parent tetrabenzoporphyrin **Pt-12** (λ_em_ = 770 nm) could be obtained, resulting from a stabilization of the LUMO energy level of the porphyrin ring. Conversely, a bathochromic shift of only 60 nm was observed for **Pt-24** (λ_em_ = 830 nm) which comprises of two nitrogen atoms, assigned to a localization of the triplet state only on the half-moiety of the porphyrin cycle comprising one nitrogen atom and one meso-carbon atom. Therefore, it can be concluded that the introduction of second nitrogen has a detrimental effect on the emission wavelength. When tested in a standard device structure (ITO/PEDOT:PSS/NPD (30 nm)/TAPC (10 nm)/Alq_3_:4% dopant (25 nm)/BCP (40 nm)/LiF/Al), an EQE of 2.8 and 1.5% were, respectively, obtained for **Pt-23** and **Pt-24**. As an interesting feature, the full width at half maximum (FWHM) was narrow (27 nm for **Pt-23** contrarily to 40 nm for the reference **Pt-12**), ensuring that the emission only occurs in the NIR.

### 2.2. Iridium Complexes

**Figure 3:**
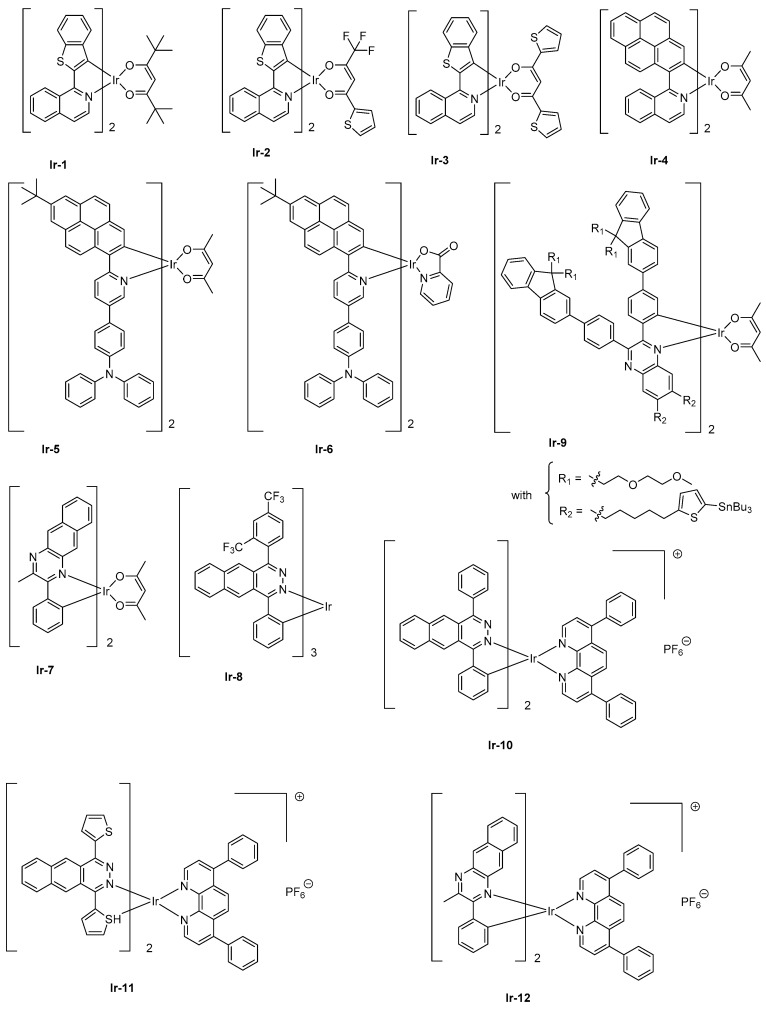
Ir-based near-infrared (NIR) emitters for light-emitting diodes.

Iridium complexes have long been studied for the design of visible light electroluminescent devices, and cationic, anionic or neutral complexes have been examined for this purpose [70,71,72]. Only recently, iridium complexes have been explored to elaborate NIR OLEDs. Contrarily to platinum complexes that possess a square planar structure and long-living excited state lifetimes favorable to T-T annihilation and facilitating the efficiency roll-off by increasing the current density, iridium complexes differ by their octahedral geometries and their reduced excited state lifetimes. Iridium is also a cheaper metal than platinum, so d^6^ iridium complexes have been identified as a viable alternative to platinum complexes. Here again and capitalizing on the strategies developed for platinum complexes, the efficient method to induce a significant bathochromic shift of the emission and to elongate the π-conjugation of the cyclometalated ligands of iridium complexes and the introduction of electron-rich heteroaromatic rings was applied [73]. To illustrate this, the replacement of a 2-phenylpyridine by a 2-naphthylisoquinoline ligand could shift the emission spectrum of a *tris*(cyclometalated)iridium complex from more than 100 nm [74,75]. Alternatively, a destabilization of the energy levels can be achieved by use of an ancillary ligand, but only a slight shift of the emission can be obtained with this strategy (10–15 nm) [76,77,78]. Besides, the combination of the two approaches proved to be effective for developing NIR emitters based on iridium. This strategy was notably applied for the design of a family of [Ir(iqbt)_2_L] complexes where an electron-rich cyclometalated ligand, that is, iqbt which stands for 1-(benzo[*b*]thiophen-2-yl)isoquinoline was combined with three different ancillary ligands, namely 2,2,6,6-tetramethyl-3,5-heptanedione (Hdpm) (furnishing **Ir-1**), 2-thienoyltrifluoroacetone (Htta) (furnishing **Ir-2**), and 1,3-di(thiophen-2-yl)propane-1,3-dione (Hdtdk) (furnishing **Ir-3**) (See Figure 3) [79]. Precisely, the last two ancillary ligands have been selected for the presence of electron-rich groups, that is, thiophene units. In solution, **Ir-1-Ir-3** displayed an emission at 710, 704, and 707 nm, respectively, consistent with the electronic enrichment of the ancillary ligand. From these results, the weak influence of the chemical modification of the ancillary ligand and the introduction of thiophene units, the bathochromic shift of the emission being of only 3 nm between complexes **Ir-2** and **Ir-3**, can also be concluded. Conversely, if the photophysical properties of **Ir-1** and **Ir-3** were almost identical (PLQY = 0.16 and 0.14, excited state lifetimes = 1.40 μs and 1.44 μs for complexes **Ir-1** and **Ir-3**, respectively), a significant decrease was observed for complex **Ir-2** (0.07 and 0.72 μs). Examination of the non-radiative decay rate evidenced this constant to be two times higher than that determined for complexes **Ir-1** and **Ir-3** whereas similar excited state lifetimes could be measured for all complexes at 77K. Therefore, it was concluded that specifically, for complex **Ir-2**, a non-radiative deexcitation pathway was thermally favored at room temperature. The EL performances of complexes **Ir-1- Ir-3** in solution-processed devices followed the trend observed for the photophysical properties, complexes **Ir-1** and **Ir-3** furnishing the highest EQE (3.07 and 2.44%, respectively) whereas the performances of complex **Ir-2** were clearly behind (1.28%). A NIR emission was detected for all complexes, the emission wavelength ranging from 714 nm for complexes **Ir-1** and **Ir-3** to 709 nm for complex **Ir-2** (see Table 2). As a positive point, all devices showed a negligible efficiency roll-off, lower than 10% between 0 and 1 W·sr^−1^·m^−2^. There are numerous examples of heteroleptic iridium complexes with cyclometalated ligands of extended poly-aromaticity to produce NIR emitting materials in the literature. For instance, the introduction of pyrene units into a cyclometalated ligand [31] or anthracene units [80] can be cited as examples. However, all these NIR emitters have not been designed for OLEDs applications, and some of these structures have been prepared for biological applications [80].

While coming back to **Ir-4**, a NIR EL emission at 720 nm and an EQE of 0.27% could be obtained with this complex when tested as triplet emitter for solution-processed OLEDs (See Table 2). A two-fold enhancement of EQE could even be obtained by introducing a hole-transport triphenylamine (**Ir-5**) at the peripheral side of the pyrene-based cyclometalated ligand [81]. EQE could be improved to 0.56% while doping the emissive layer at 4 wt % with **Ir-5**. A NIR emission extending from 697 nm (main peak) to 764 nm (shoulder) could also be determined, mirroring the PL spectrum.

Enhancement of the EL performance can be assigned not only to the presence of the hole-transport unit onto the complex facilitating the charge transportation but also to the introduction of bulky substituents in charge to drastically reduce the aggregation in the solid state. Finally, the replacement of the acac ligand of **Ir-5** by a picolinate ligand (pic) in **Ir-6** does not significantly alter the EL spectrum (main peak at 698 nm with a shoulder at 762 nm), and a higher EQE could be obtained with this value peaking at 1.29% for vacuum-processed OLEDs [82]. While coming back to complexes comprising of acac ligand, the heteroleptic complex **Ir-7** comprising of cyclometalated ligand 2-methyl-3-phenylbenzo[*g*]quinoxaline (mpbqx-g) could emit at 777 nm with a shoulder at 850 nm [83]. An EQE of 2.2% was obtained while doping the emissive layer at 20 wt %. A low-efficiency roll-off was also evidenced resulting from a relatively short phosphorescence lifetime (0.28 μs). Based on the extended π-conjugated benzo[*g*]phthalazine ligand, which is of similar structure as that of mpbqx-g, the homoleptic complex fabricated with this ligand, that is, **Ir-8**, could exhibit a peak emission at 760 nm with an EQE of 4.5% for evaporated OLEDs and a dopant concentration of 12 wt % [84]. By developing more sophisticated cyclometalated ligands, EQE of **Ir-9** could be increased up to 3.4% for an EL emission at 702 nm and devices prepared by solution process. As specificity, this complex has been designed with bulky peripheral substituents so that the complex is itself “encapsulated” by its own substituents, reducing the possible intermolecular interactions, T-T annihilation and addressing the efficiency roll-off issue. To overcome the problems inherent with polyaromatic structures, that is, the low solubility, alkyl chains were introduced onto the fluorene units. Authors also evidenced light emission to originate from charge trapping by the complex, resulting in a significant increase of the driving voltage upon increase of the dopant concentration. Concerning the low-efficiency roll-off, authors attributed this specificity to the short-excited state lifetime of the complex and the bulkiness of the peripheral groups.

Finally, iridium complexes can also be synthesized under the cationic form, and a few examples of NIR cationic complexes have been reported in the literature. As a drawback, cationic iridium complexes can’t be sublimed and use of this emitter, therefore, imposes the elaboration of devices by solution-process. As first examples of cationic complexes, **Ir-10** and **Ir-11** could produce a true NIR emission at 715/788 and 791 nm with EQEs of 0.50 and 0.34%, respectively [85]. In these structures, the benzo[*g*]phthalazine ligand could induce a much stronger Ir–N bond than the benzo[*g*]quinoline ligands, providing emitters with higher thermal stability. The insensitivity of OLEDs to the current density was also demonstrated, addressing the efficiency roll-off issue. Finally, **Ir-12** is another cationic complex of interest [86]. Here again, use of 2-methyl-3-phenylbenzo[g]quinoxaline (mpbqx) as the ancillary ligand enabled to produce a true NIR emission (753 nm) together with an acceptable EQE (0.30%).

**Figure 4:**
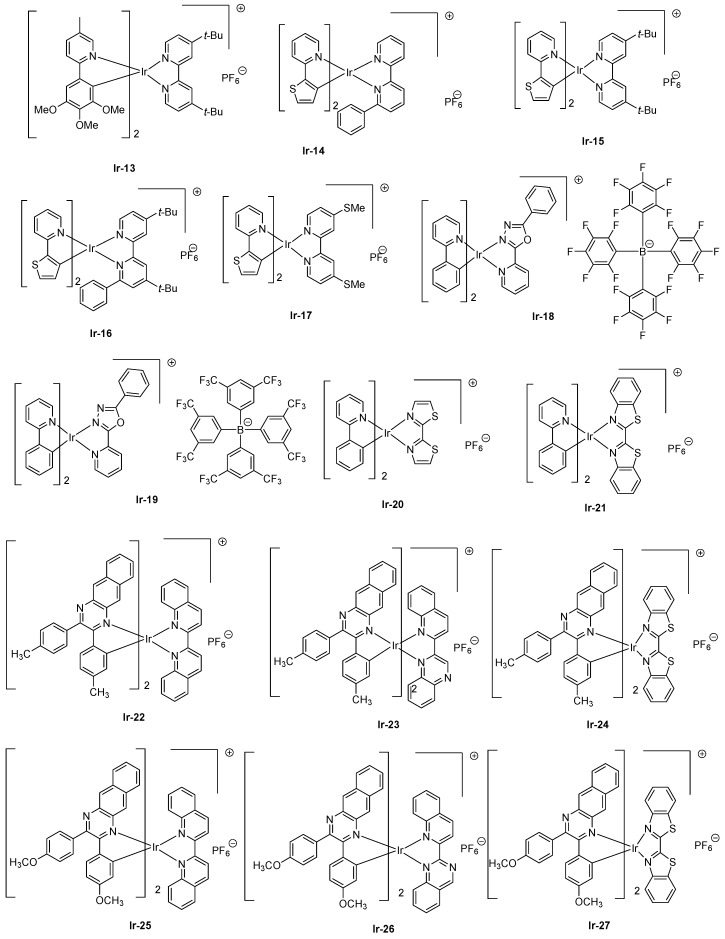
Ir-based near-infrared (NIR) emitters for light-emitting diodes and light-emitting electrochemical cells.

Concerning cationic iridium complexes, several strategies have been developed over the years to red-shift their emissions and investigate their incorporation into light-emitting electrochemical cells (LECs). As specificity, LECs differ from OLEDs by the presence of mobile ions within the emissive layer so that a delay occurs between the application of a driving voltage and light emission [87]. Contrarily to OLEDs where their characterizations are realized by sweeping the driving voltage between zero and a maximum voltage defined by the manipulator in order to determine their current-voltage-luminance (I-V-L) characteristics, LECs require, prior to light emission, a step consisting of doping both interfaces to facilitate charges injection. Doping of interfaces can be obtained by applying a constant voltage, enabling ions pair separation, and the migration of ions at both interfaces, reducing the energy barrier to inject electrons and holes. Consequently, a delay occurs between turn-on time and light emission due to the time required to form the p-n junction. While coming to the light emitting materials, and considering that for iridium complexes the HOMO energy level is centered on the cyclometalated ligands and the metal center, several studies were devoted to destabilize the HOMO energy level by mean of electron-releasing groups, such as methoxy groups (**Ir-13**) [88], electron-rich groups, such as thiophene (**Ir-14-Ir-17**) [89], or extended polyaromatic groups, such as benzo[*g*]quinoline (**Ir-12**) (see Figure 3 and Figure 4). As specificity, by applying a driving voltage of 4V to LECs containing **Ir-13**, the maximum luminance was achieved after operating LECs for one hour (18 cd/m²), and a half-life of two hours was also determined for these devices. An extremely low EQE of 0.05% was obtained. Interestingly, LECs emit at 650 nm, with a broad emission band extending from 550 to 850 nm. Similar behavior was observed with **Ir14-Ir-17**, for which a maximum emission was detected at ca. 600 nm for all complexes. However, the emission was also broad, the electroluminescence (EL) peaks extending from 550 to 800 nm. Contrarily to **Ir-13** for which a short device lifetime was determined, half-lives of 101 and 9.7 h were obtained with **Ir-14** and **Ir-16**, respectively, possessing a 6-phenyl-2,2’-bipyridine ligand. This ligand is notably extensively used to improve the chemical stability of iridium complexes by generating π-π interactions between the cyclometalated ligands and the ancillary ligand [90]. Another strategy commonly used to decrease the HOMO-LUMO consists of stabilizing the LUMO energy level, which is achievable upon extending the π-conjugation of the ancillary ligand. In this context, OLEDs could even be prepared with **Ir-18** and **Ir-19** which are proved to be sublimable cationic complexes [91]. However, the limitation of this second strategy is obvious, since an emission at 608 nm was found for the two complexes, the emission peak extending from 500 to 800 nm. 2,2′-Bithiazoles and 2,2′-bibenzo[*d*]thiazoles that belong to a new family of ancillary ligands prove to be a more efficient strategy to tune the LUMO energy level of iridium complexes [92]. By extending the aromaticity of the ancillary ligand in **Ir-21** relative to that of **Ir-20**, the EL peak could be shifted from 661 to 705 nm for **Ir-20** and **Ir-21**, respectively. However, for the two complexes, EQE obtained for OLEDs remained low, peaking at 0.13 and 0.33% for **Ir-20** and **Ir-21**, respectively (see Table 3). Recently, a breakthrough has been achieved by combining both the extension of aromaticity of the ancillary ligands and the cyclometalated ligands [93]. To evidence the benefits of this strategy, six complexes **Ir-22-Ir-27** were synthesized. Almost similar photoluminescence properties were found for the six complexes, varying between 827 for **Ir-26** to 852 nm for **Ir-22**. A near-infrared emission detected beyond 800 nm could be determined for all complexes, irrespective of the substitution pattern or the ancillary ligands. However, the most red-shifted emission was found for complexes comprising 2-(quinolin-2-yl)quinazoline as the ancillary ligand (849 and 846 nm for **Ir-24** and **Ir-25**, respectively) or 2,2′-biquinoline (852 and 840 nm for **Ir-22** and **Ir-23**, respectively). Among all synthesized complexes, only **Ir-24** and **Ir-27** were tested as solid-state emitters for LECs. In a conventional device stacking, LECs fabricated with **Ir-24** could emit at 882 nm whereas the emission of **Ir-27**-based LECs was blue-shifted compared to that of **Ir-24**-based devices, peaking at 790 nm. If the electron-to-photon conversion remained low with these complexes, the device lifetime was extremely low, and the overall lifetimes of LECs of approximately 2 min before a complete and irreversible degradation of the emitters was evidenced.

### 2.3. Ruthenium Complexes

**Figure 5:**
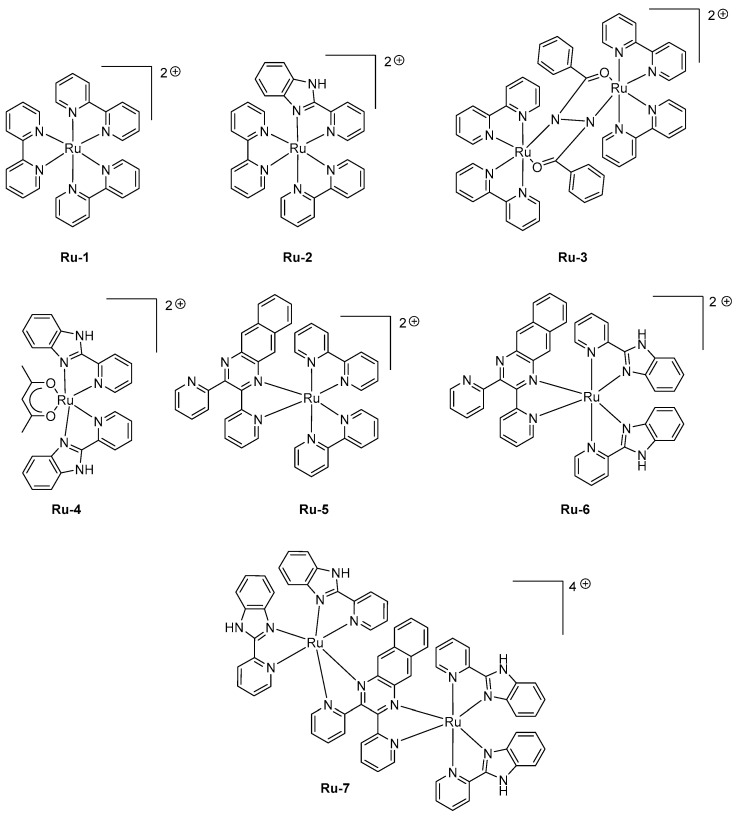
Ru-based near-infrared (NIR) emitters for light-emitting electrochemical cells.

Ruthenium complexes have also been extensively studied for the design of LECs as the high molecular weight of these complexes is a major impediment for the design of OLEDs by vacuum processes. Historically, ruthenium complexes have been the first family of triplet emitters to be tested as light-emitting materials for solid-state devices, but their relative long excited state lifetimes on the basis of numerous quenching processes (triplet-triplet (T-T) annihilation, triplet polaron annihilation) and the weak color tunability have rapidly discarded these complexes in favor of iridium complexes [94]. The first examples of Ru complexes exhibiting a near-infrared emission were reported in 2008, and mononuclear and di-nuclear complexes were indifferently investigated in this study [95]. Seven complexes **Ru-1-Ru-7** were designed, varying by the nature of the ligands (see Figure 5). While examining their photoluminescence properties, a red-shift was clearly observed while replacing the classical 2,2′-bipyridine by 2-(2-pyridyl)benzimidazole and finally 2,3-*bis*(2-pyridyl)benzoquinoxaline.

Thus, a red-shift of the PL emission from 650 nm for **Ru-1** to 1040 nm for **Ru-7** could be obtained. When tested in LECs with a standard device configuration of ITO/**Ru-1-Ru-7** (100 nm)/Au, a contribution in the near-infrared region could be found for all emitters, the EL emission peaking at 630 nm for **Ru-1** to 1040 nm for **Ru-7**. Good accordance between the EL and PL spectra could be found for all complexes. If **Ru-7** gave LECs with the most red-shifted emission, the maximum luminance of external quantum efficiency could not be determined for this complex due to the low light intensity. Interestingly, all complexes (i.e., **Ru-4**, **Ru-6**) comprising 2-(2-pyridyl)-benzimidazole as the ligand furnished devices that could only be driven at higher voltage compared to that measured with the other complexes. A turn-on time varying from a few seconds to a hundred of seconds could be determined for all complexes, depending on the applied voltage. The fastest response time was obtained for **Ru-3**, existing under the form of a mixed valence state (Ru^2+^/Ru^3+^) during the doping step, facilitating charge transport.

**Figure 6:**
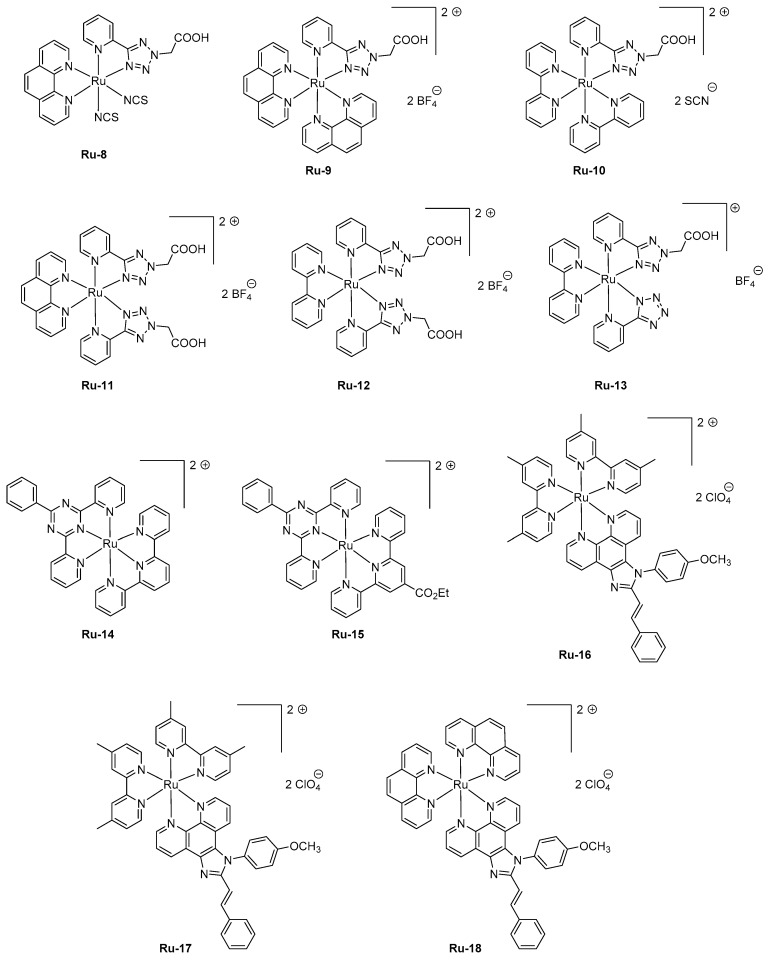
Ru-based near-infrared (NIR) emitters for light-emitting electrochemical cells.

As previously mentioned, the device-stacking is an important parameter influencing the overall performance. A magistral demonstration was done with **Ru-1**, revisited in the context of a polymer-based LEC [96]. In this work, poly(vinyl)alcohol was used as the host material, and maximum luminance of 6.89 cd/m² could be obtained while maintaining the EL emission at 620 nm and introducing a reduced graphene oxide layer between the anode and the emissive layer. Parallel to the improvement of the electron-to-photon conversion, a severe improvement of the device stability was obtained, enhanced from a few minutes in the former study to 37 min in this work by using the following device structure ITO/reduced graphene oxide (rGO)/**Ru-1**/Ag. If the rGO layer was beneficial concerning the device stability, the performance could be even improved by removing this layer, enabling LECs to reach a peak efficiency of 14.42 cd/m². In 2016, an unusual ligand, namely 2-(5-(pyridin-2-yl)-2*H*-tetrazol-2-yl) acetic acid, was used as the key ligand for the design of a series of six complexes **Ru-8-Ru-13** (see Figure 6) [97]. Compared to the former series **Ru-1-Ru-7** comprising 2-(2-pyridyl)benzimidazole or 2,3-*bis*(2-pyridyl)-benzoquinoxaline, a decrease of the HOMO-LUMO gap was less efficient since EL emissions ranging from 568 nm for **Ru-13** to 612 nm for **Ru-8** were determined for LECs comprising these emitters. Noticeably, the EL emission was broad so that a contribution in the NIR region could be found for all complexes. Considering that numerous combinations of ligands were used in this study, several conclusions could be established. Thus, the 2-pyridine (1*H*-tetrazol-5-yl) ligand in **Ru-13** greatly contributed to blue-shift the EL emission (568 nm) compared to that of **Ru-12** comprising 2-(5-(pyridin-2-yl)-2H-tetrazol-2-yl)acetic acid (600 nm). Similarly, the choice of the ancillary ligand also proved to be crucial. A comparison between **Ru-11** and **Ru-12** differing by a phenanthroline or a bipyridine ancillary ligand evidenced a difference of the maximum EL emission to vary from 25 nm. To improve the device stability, a four-layer LEC structure was used, using the following device stacking: ITO/PEDOT-PSS/PVK/Ru complex/PBD/Al. Notably, the emissive layer was separated from the electrodes by the introduction of a hole-injection layer (PEDOT:PSS) and a hole-transport layer (poly(*N*-vinyl)carbazole (PVK)) at the anode side and by an electron transport layer (2-(4-*tert*-butylphenyl)-5-(4-biphenylyl)-1,3,4-oxadiazole PBD) at the cathode interface to avoid electrons and holes to drift at both interfaces and initiate quenching processes. PVK is notably extensively used for the design of solution-processed devices due to its ability to drastically reduce the surface roughness of the indium-tin-oxide (ITO) anode by its remarkable film-forming ability [98,99,100]. Influence of the counter-anion on the device stability was also examined. Concerning this point, the best stability was found with all emitters containing the tetrafluoroborate anion. On the opposite, the less stable devices were fabricated with emitters comprising thiocyanate as the anion (**Ru-8**, **Ru-10**), the latter being converted to cyanide anion by sulfur elimination during device operation [101,102]. For **Ru-9**, **Ru-11-Ru-13**, device stability higher than 20 h could be found, demonstrating 2-(5-(pyridin-2-yl)-2*H*-tetrazol-2-yl) acetic acid to enable the elaboration of remarkably stable complexes, despite the presence of the acetic acid group. Concerning the device stability, remarkable results were obtained with two heteroleptic ruthenium *bis*-chelate complexes comprising substituted tridentate 2-phenyl-4,6-dipyridin-2-yl-1,3,5-triazine ligands [103]. Choice of this ligand was dictated by a comparison established with the well-known terpyridine ligand extensively used for the design of ruthenium complexes. Notably, numerous works on 2-phenyl-4,6-dipyridin-2-yl-1,3,5-triazine ligands revealed the ruthenium complexes fabricated with this ligand to exhibit higher photoluminescence quantum yields and elongated excited state lifetimes compared to their analogs based on terpyridine [104,105,106,107]. To get a luminescence at room temperature, two complexes were designed, that is, **Ru-14** and **Ru-15** varying by the presence of the electron-withdrawing ester group. Photoluminescence of the two complexes only slightly varies, originating from the ^3^MLCT state and peaking at 723 and 717 nm for **Ru-14** and **Ru-15**, respectively. To get emissive layers with sufficiently smooth properties, the two complexes were mixed with 20% poly(methyl methacrylate) (PMMA). When tested in a conventional device structure ITO/PEDOT:PSS**/Ru-14** or **Ru-15** :PMMA/Al, presence of the saturated polymer within the emissive layer resulted in devices with low light output, around 0.6 μW, and requiring several hours to reach the maximum luminance (9 and 37 h for **Ru-14** and **Ru-15**, respectively), indicative of a reduced ion mobility in the PMMA layer in both cases. The most stable devices were obtained with **Ru-15**, the time to reach half of the initial luminance being of 360 h, contrarily to 120 h, for **Ru-14**-based LECs. For the two complexes, maximum EQEs remained low, peaking at 0.005%. Performance of LECs can also be improved by providing more balanced charge transportation within the emissive layer. This parameter was examined with a series of three complexes **Ru-16-Ru-18** where an ambipolar charge transportation ability was provided by attaching a phenanthroimidazole ligand [108]. As an interesting point and in addition to the improvement of the charge transportation, use of an ancillary ligand with extended aromaticity both contribute to reducing the HOMO-LUMO gap and red-shift the PL emission. While examining the PL emissions in solution and in thin films, a major red-shift of the maximum emission was observed for **Ru-17**, shifting from 630 nm in solution to 700 nm in thin films, indicating a severe aggregation in the solid state. Conversely, a more moderate shift was observed for **Ru-16** and **Ru-18**, shifting from 609 and 594 nm in solution to 628 and 631 nm in the solid state for **Ru-16** and **Ru-18**, respectively. LECs fabricated with **Ru-16-Ru-18** were prepared with an unusual cathode, namely a Ga:In alloy that avoids the deposition of this electrode at high temperature. **Ru-16-Ru-18**-based LECs clearly evidenced the red-shift of the EL emission compared to the reference Ru(bpy)_3_^2+^-based LECs. If the EL emission is detected at 632 nm for Ru(bpy)_3_^2+^-based LECs, the EL emissions were, respectively, observed at 664, 695, and 644 nm for **Ru-16**, **Ru-17,** and **Ru-18**-based LECs. The emission peaks were broad for LECs, extending from 550 nm until 900 nm. Considering that the EL emission of LECs is mostly centered in the visible range, EQEs as high as 1.40, 0.93, and 1.15% were calculated for **Ru-16** and **Ru-18,** respectively.

**Figure 7:**
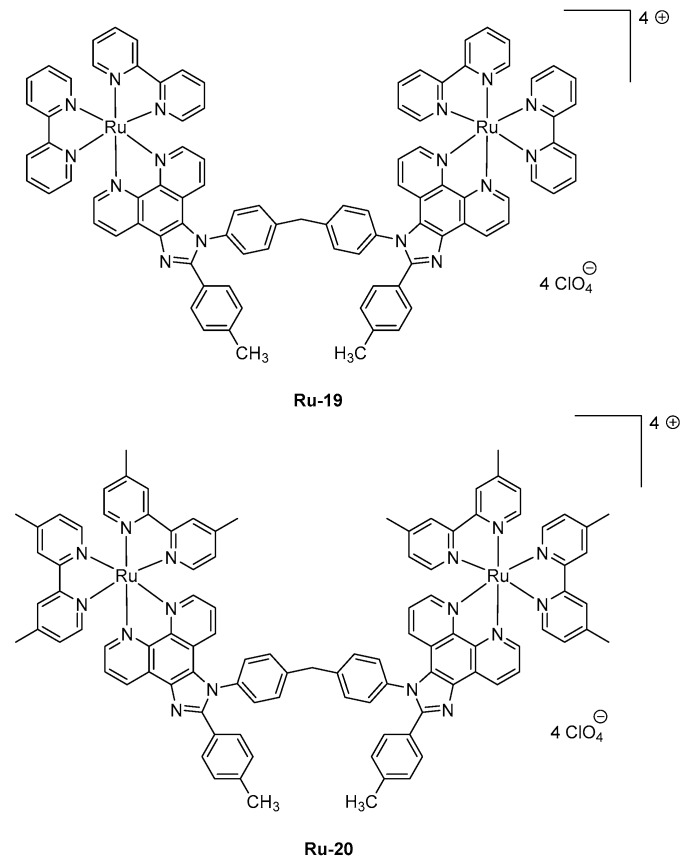
Ru-based near-infrared (NIR) emitters for light-emitting electrochemical cells.

Examination of the device lifetimes revealed the three complexes to give LECs of comparable stability, in the order of 1000 min, corresponding to the time required to reach half of the initial luminance. The last strategy developed to induce a NIR emission is the use of polynuclear complexes. This strategy is quite unusual considering the difficulties of synthesis of such complexes and the problems of solubility encountered with these polymetallic structures. This work is notably justified by the fact that LECs based on complexes comprising phenanthroimidazole ligands often lack the acceptable device stability for future applications, which could be improved by using di-nuclear complexes [109,110]. However, examination of LECs characteristics revealed that this challenge could not overcome with **Ru-19** and **Ru-20**, the time for LECs to reach half of the initial luminance being of only 539 and 1104 s for **Ru-19** and **Ru-20**-based devices, respectively (See Figure 7 and Table 4) [111]. From this work, it can be, therefore, concluded that the design of polynuclear Ru-complexes requiring hard work from the synthetic point of view is useless and non-adapted for the design of long-living LECs.

### 2.4. Lanthanide Complexes

Rapidly after the discovery of the electroluminescence process with Alq_3_, numerous works have been devoted to examining the EL properties of complexes comprising of rare earth metals. Due to the presence of 4f electrons, numerous electrons, and energetically close levels, these complexes were immediately identified as appealing candidates for optical transitions in the near-infrared region [112,113]. Indeed, lanthanide complexes are characterized by sharp EL emission bands due to the 4f electrons of the cationic center. Resulting from the important size of the metal center, complexes of rare Earth metals also exhibit relatively flexible coordination geometries, enabling to largely tune their optoelectronic properties. By combining various β-diketonates and ancillary ligands, geometries of these flexible complexes could be optimized so that the PLQYs of lanthanide complexes are greatly improved [114,115,116,117]. Among ligands, β-diketones are the most versatile ones, by their facile substitutions, their strong coordination ability, and π–π* transitions located in the UV region. When combined with O^N ancillary ligands, the coordination sphere around the lanthanide center is complete so that there is no space for high energy O–H or C–H oscillations of solvent molecules [114,115,116,117,118,119,120,121,122,123]. Indeed, lanthanide complexes are highly sensitive to their environment.

**Figure 8:**
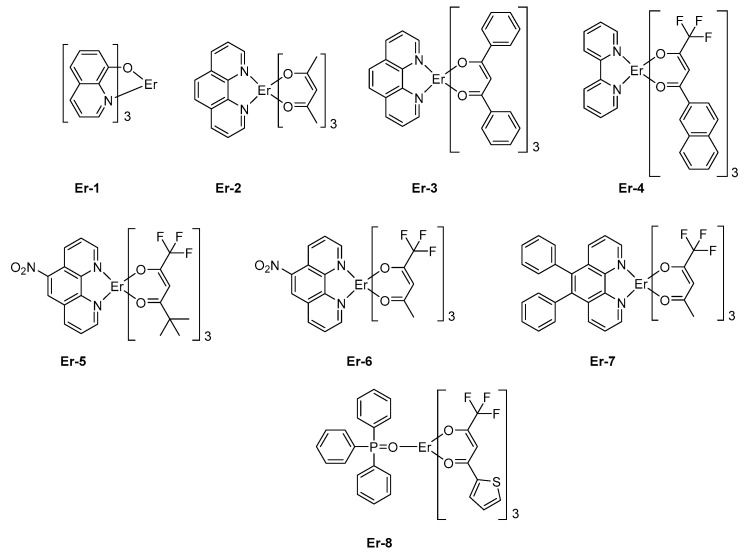
Er-based near-infrared (NIR) emitters for light-emitting diodes.

Parallel to this, the asymmetric coordination geometries around the metal center are known to give strong emission efficiencies. Among all possible metal centers of Rare Earth, the optical transition of the trivalent erbium ion Er^3+^, that is, ^4^I_3/2_ → ^4^I_15/2_ occurs at 1.5 μm which corresponds to the standard telecommunications windows, rendering this metal of crucial interest for both civil and military applications. For instance, erbium *tris*(8-hydroxyquinolate) **Er-1** was used for the design of the early OLEDs emitting at 1.54 μm [124,125]. No quantification of the light emission properties was provided, and simple device architectures were used as exemplified by the following structure: ITO/TPD (50 nm)/**Er-1** (60 nm)/Al and others [126]. In 2000, Sun and coworkers mixed another trivalent Er complex, **Er-2**, in PVK as the host polymer, and OLEDs emitting at 1.54 μm were also obtained [127]. However, OLEDs remained single-layered devices, limiting the EL efficiencies. Rapidly, the device architecture was improved, and the first attempt to optimize the device-stacking was carried out in 2010 by Wei et al. with **Er-3** [128]. Top-emitting devices were fabricated since OLEDs were elaborated on Si wafers. The geometry of Er complexes can greatly affect the emissive properties, and over the years, a great deal of efforts has been devoted to achieving the most favorable geometry. This is notably the case for erbium (III) β-diketonate complexes [113,114,115,116,117,118,119,120,121,122,123,124]. Fluorinated β-diketonate complexes with N^N-donor ancillary ligands have notably been developed for their remarkable solubility, allowing the design of solution-processed OLEDs with **Er-4** [129] or **Er-5** (See Figure 8) [130]. Fluorination of β-diketonate ligands is also an effective way to improve the solubility of complexes without significantly affecting the triplet energy level of the β-diketonate used as sensitizing ligands [131]. Solution-processed OLEDs fabricated with **Er-5** showed the typical energy transfer from the organic ligand to the central Er (III) ion, with an emission detected at 1535 nm corresponding to a ^4^I_13/2_ → ^4^I_15/2_ transition. Interestingly, devices exhibited a low turn-on voltage of 7V [129] or 8V [132], depending on the study.

These values are comparable to that reported for other octacoordinated Er complexes, such as **Er-6** [133] or **Er-4** [130]. However, in the case of **Er-7**, the turn-on voltage could be lowered to 4 V, but a dramatic decrease of the maximum brightness was also demonstrated, the latter being three times lower than that of **Er-6** (see Table 5). Finally, by using a neutral triphenylphosphine oxide as the ancillary ligand, a dramatic impact on both the turn-on voltage (14.0 V) and the maximum irradiance (0.069 mW/cm²) could be evidenced with **Er-8** as the emitter [134]. Choice of the metal center introduced in the lanthanide complexes is of crucial importance as it governs the emission wavelength of OLEDs. The second most widely studied metal for the design of NIR emitters is neodymium. In this case, emission of OLEDs is centered at 1065 nm. The first report mentioning the observation of electroluminescence from a neodymium complex was reported in 1999 by Kawamura et al. [135].

**Figure 9:**
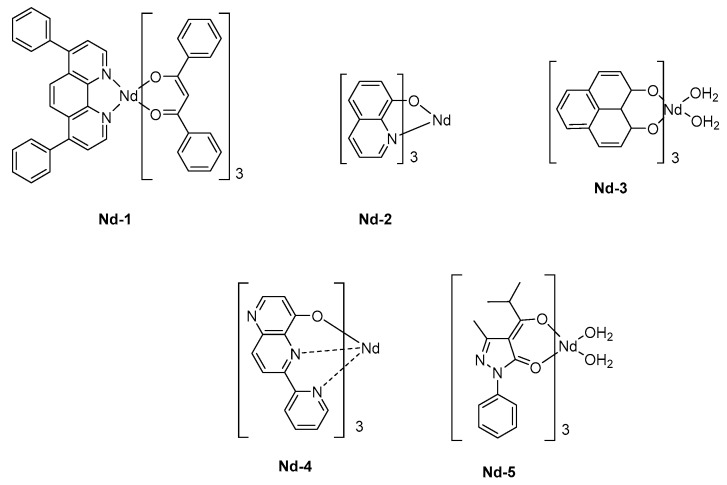
Nd-based near-infrared (NIR) emitters for light-emitting diodes.

A triple layered device was then used, comprising a hole and an electron transport layer, thus favoring the charge recombination within the emissive layer. Capitalizing on the results obtained by Tang and VanSlyke, Alq_3_ was used as the electron-transport layer. The ancillary ligand of **Nd-1** was 4,7-diphenyl-1,10-phenanthroline (bath), selected for its excellent charge transport ability whereas the sensitization of the neodymium cation was ensured by dibenzoylmethane ligands (see Figure 9). Upon application of a driving voltage of 19V, clear electroluminescence of the complex in the NIR region was detected, producing three sharp emission bands at 890, 1070, and 1350 nm corresponding to ^4^F_3/2_ → ^4^I_9/2_, ^4^F_3/2_→ ^4^I_11/2_, and ^4^F_3/2_ → ^4^I_13/2_ transitions, respectively. As a drawback, a significant peak corresponding to the green EL of Alq_3_ could be detected at a high driving voltage so that the intensity of the visible emission could become comparable to that detected in the NIR region. By replacing Alq_3_ by a hole-blocking layer (BCP), a pure emission of **Nd-1** could be obtained by confining holes within the emissive layer [136]. The chirality of complexes can alter the emission wavelength of OLEDs, and the influence of the isomers of a same complex on the EL characteristics was demonstrated in the early work of Khreis et al. (**Nd-2**) [137]. In this work, authors could demonstrate the thermal isomerization of one isomer to another one, resulting in the enrichment of the emissive layer with one isomer. However, if the comparison of the PL spectra of the powder and thin films could evidence the phenomenon, authors could not determine which isomer could be rearranged thermally. To produce a NIR emission, the sensitization of the Nd^3+^ cation is crucial, and some authors selected 1*H*-phenalen-1-one as the ligand due to its common use in biology as singlet oxygen sensitizer. The possibility to sensitize the Nd^3+^ cation was demonstrated, and the main peak at 1065 nm could be detected for OLEDs fabricated with **Nd-3** [138]. An EQE of 0.007% could be determined for these polymer LEDs. These performances could be improved by sensitizing the cation with a tridentate ligand, that is, 6-(pyridin-2-yl)-1,5-naphthyridin-4-ol, and an EQE of 0.019% could be reached (**Nd-4**) [139]. Finally, the best EQE was obtained in 2010, by co-depositing an iridium complex with the Nd^3+^ complex **Nd-5** (See Table 6) [140]. Benefits of a triplet sensitizer were demonstrated since the maximum EQE could reach 0.3%. However, the pertinence of the strategy can be still discussed, with regards to the high cost of iridium complexes used as the sensitizer.

The asymmetry of the structures of lanthanide complexes is well-reported to favor more the radiative deexcitation pathways compared to the symmetric complexes [114,119]. If the former lanthanides complexes (erbium, neodymium) were developed for telecommunication and laser applications, emission of ytterbium complexes is centered around 1000 nm, and these complexes thus found applications for photodynamic therapy and/or detection of tumors [141,142]. As specificity, ytterbium complexes exhibit slightly higher PLQYs than the other lanthanides and longer-living excited state lifetime in the microsecond range. Yb^3+^ also possesses 33 electrons in its 4f orbitals, and a pure emission around 980 nm can be easily obtained resulting from a transition from the ground state ^2^F_5/2_ to the excited state ^2^F_7/2_. Based on the observation that asymmetric complexes were more emissive than the symmetric ones, ytterbium complexes displaying an asymmetry structure were designed as emitters for OLEDs. Proof of concept that NIR OLEDs could be fabricated with a ytterbium complex was done in 2000 by Kawamura et al. [143].

**Figure 10:**
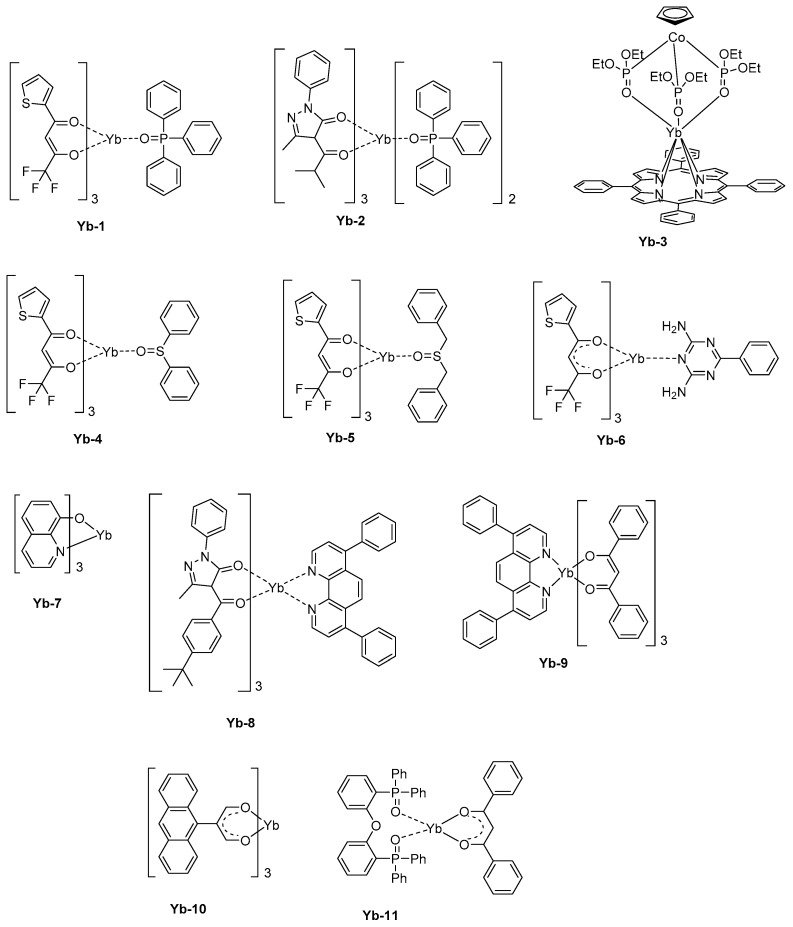
Yb-based near-infrared (NIR) emitters for light-emitting diodes.

In a basic device structure (hole-transport layer/emissive layer/electron transport layer), a pure emission of Yb^3+^ could be electrogenerated. Thus, **Yb-1** that comprises a triphenylphosphine oxide as the ancillary ligand and thenoyltrifluoroacetylacetone as the monoanionic ligand could furnish a maximum irradiance of 19.29 μW/cm² at 15 V [144]. This value is significantly higher than that reported for **Yb-2** (1.47 μW/cm² at 17.8 V) [145] or **Yb-3** [146] (0.6 μW/cm² at 15.7 V) (see Figure 10). It has to be noticed that for the last complex, that is, **Yb-3**, the emissive layer was made of the metal complex blended with the insulating polystyrene polymer, what was not favorable for charge transportation. Later, the same author blended **Yb-3** with a poly(paraphenylene) polymer, improving the charge transport and reaching a maximum irradiance of 10 μW/cm² at 9 V [30,147,148]. While coming back to **Yb-1**, the EL emission detected at 980 nm corresponds to the ^2^F_5/2_→^2^F_7/2_ transition. However, two other broad emissions could be detected at 410 and 600 nm, assigned to the electroplex formation at the interface between organic layers. The thenoyltrifluoroacetylacetone is a promising ligand for the design of highly emissive Yb^3+^ complexes, and another asymmetric seven-coordinate complex with a square antiprism (C_4v_) geometry, that is, **Yb-4,** can be cited as an efficient NIR complex [149]. In this work, a series of three ancillary ligands were examined, namely diphenyl sulphoxide, dibenzoyl sulphoxide, and benzoguanamine. Diphenyl sulphoxide was found to complete the coordination sphere around the Yb^3+^ cation the most efficiently.

By using a device structure which is classically used for visible LEDs (ITO)/β-NPB (25 nm)/[Yb-complexes] (10 wt %):TcTa (40 nm)/BCP (15 nm)/Alq_3_ (10 nm)/LiF (0.5 nm)/Al), a maximum irradiance of 22.48 μW/cm² could be obtained with **Yb-4** whereas this value was reduced to 12.13 μW/cm² and 9.60 μW/cm² for **Yb-5** and **Yb-6,** respectively (See Figure 10 and Table 7). The order of the maximum irradiances follows those of the PLQYs, the EL efficiency being proportional to the PLQYs. Here again, two emissions at 410 and 600 nm could be detected, once again assigned to the formation of electroplex at the organic interface. Charge recombination and energy transfer on the organic ligand are well-known and were notably observed for a complex, such as **Yb-7** [150]. To favor the charge recombination within the emissive layer, a lot of efforts has been devoted to the fabrication of OLEDs comprising double emissive layers. This is the case with **Yb-2** and **Yb-8** that were both introduced within the emissive layer [145]. Recombination of holes and electrons within the emissive layer was facilitated by the hole-transport ability of **Yb-8** and the electron-transport ability of **Yb-2**. Consequently, electrons and holes could recombine at the **Yb-8**/**Yb-2** interface, and a pure emission of Yb^3+^ could be obtained. A similar strategy was also developed only for **Yb-9**, with a **Yb-9**:TPD/**Yb-9** bilayer [151]. In this early work published in 2001, no quantification of the maximum irradiance was done. However, the comparison carried out with devices comprising a single emissive layer evidenced a lower NIR emission intensity at comparable driving voltage. The simplification of the device fabrication constitutes a great challenge for future applications and recently, a group examined the possibility to design host-free NIR OLEDs [152].

Considering that in this specific configuration, the hole-transportation is not ensured anymore by the host but by the light-emitting materials, the emitter should exhibit good charge carrier ability. That was notably the case with **Yb-10**. When tested as an emitter in the following device-stacking: ITO/PEDOT:PSS/**Yb-10** (30 nm)/TPBi (10 nm)/Al, a low turn-on voltage of 4.0 V was determined, and an EL emission at 978 nm with a low band at 530 nm was found. By replacing 2,2′,2″-(1,3,5-benzinetriyl)-*tris*(1-phenyl-1-*H*-benzimidazole) (TPBi) acting as an electron-transport and hole-blocking material by 3-(biphenyl-4-yl)-5-(4-tertbutylphenyl)-4-phenyl-4*H*-1,2,4-triazole (TAZ), the visible EL emission could be suppressed, and an EQE of 0.14% at 14 V was determined. A further improvement was obtained by replacing Al by Ca/Al exhibiting a lower work function and facilitating electron injection. A maximum EQE of 0.21% at 12 V was thus obtained. The thermal stability of emitters during vacuum deposition is also another major concern and, in this field, a group examined the possibility to directly generate the metal complex by co-depositing the metal precursor and the ligand [153]. Using this strategy, complexes with high molecular weight can be still used for the fabrication of OLEDs. More precisely, the complex **Yb-11** was synthesized while using the ligand *bis*[2-(diphenylphosphino)phenyl]ether oxide (DPEPO) also as the host for the thermally generated complex, the latter being frequently used as host material [154,155]. A maximum EQE of 0.15% could be realized at 1.0 mA/cm².

To end this part devoted to lanthanum complexes, other metals were rarely investigated for the design of NIR OLEDs. In this field, few holmium complexes were tested, even if these complexes exhibit three main peaks at 980, 1200, and 1500 nm, the last peak corresponds to a ^5^F_5_→^5^I_6_ transition of Ho^3+^ ion, favorable to their potential applications to optical telecommunications.

**Figure 11:**
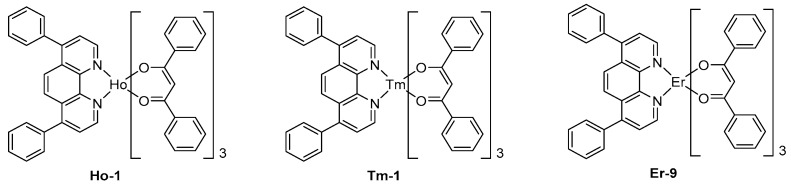
Other Lanthanum-based near-infrared (NIR) emitters for light-emitting diodes.

The EL emission of OLEDs fabricated with Ho-1 designed with standard ligands used for other lanthanum complexes with the following devices structure ITO/TPD (50 nm)/Ho-1 (50 nm)/Mg:Ag (10:1) has been proved to be adversely affected by the emission of exciplex at 660 nm, resulting from charge recombination at the TPD/EML interface (See Figure 11) [156]. As a result of this, a strong emission in the visible region competing with the NIR EL emission was evidenced. Finally, thulium complexes were only scarcely tested in NIR OLEDs, and these complexes also proved to be poor candidates for NIR emission.

Indeed, a comparison of the EL characteristics of **Tm-1**- and **Er-9**-based OLEDs evidenced the erbium complex to exhibit a much stronger emission, irrespective of the device configuration (see Figure 11) [157].

### 2.5. Osmium Complexes

**Figure 12:**
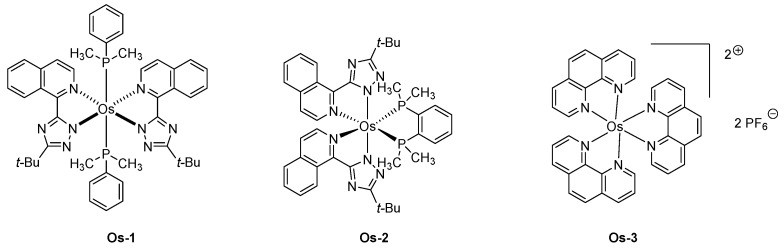
Os-based near-infrared (NIR) emitters for light-emitting diodes.

Over the years, several strategies have been developed to induce an emission centered in the NIR region. Depending on the geometry of the complex, heteroleptic complexes with cyclometalated ligands of extended conjugation were developed with iridium complexes. Conversely, the planar geometry of platinum complexes is favorable to intermolecular π-π stacking interactions in the solid state, red-shifting the emission. Concerning osmium complexes, this is the first strategy that was applied, the octahedral geometry of osmium complexes impeding π-π stacking interactions in the solid state. Therefore, the development of highly conjugated isoquinolinyl triazolate chelate was studied as a tool to red-shift the emission of osmium complexes, and EL emissions ranging from 718 to 814 nm could be obtained with **Os-1** and **Os-2** (See Figure 12) [158].

Among the two complexes, **Os-1** and **Os-2** tested in devices, OLEDs exhibiting the most red-shifted emission were obtained with complex **Os-1** (814 nm) whereas an emission at 718 nm was detected for complex **Os-2** (See Figure 12). In fact, due to the steric hindrance generated by the chelating ligands, a perpendicular arrangement of the ligand occurs, destabilizing the LUMO energy level of the isoquinolyl ligand and blue-shifting the emission. Upon optimization of the structure of the devices and the replacement of the TPBi layer by a TAZ layer, a maximum EQE of 1.5 and 2.7% was, respectively, obtained for **Os-1** and **Os-2**. It has to be noticed that the significant enhancement of the EQE for **Os-2** results from the drastically blue-shifted emission relative to that of **Os-1**. In 2005, an unexpected strategy was developed to prepare LECs, consisting of dispersed triplet emitter (**Os-3**) in an ionic ruthenium complex [159]. It has to be noticed that this approach has also been later applied to the design of OLEDs, as exemplified with the well-known Ir(ppy)_3_ hosted by various iridium complexes of wider bandgaps [160]. In the present case, Ru(bpy)_3_^2+^ was selected for its emission in the orange region, and, therefore, its energy levels were adapted to efficiently host **Os-3**. While examining the PL emission of the **Os-3**/Ru(bpy)_3_^2+^ thin films, a variation of the maximum emission with the dopant concentration was determined. Thus, emission at 675 nm was determined at 1% concentration, 695 nm at 5% concentration. Emission of doped films was determined as different from that of a pristine film of **Os-3** (710 nm), indicating solvation effects already reported in the literature for doped films [161,162]. When tested in devices, a clear shift of the EL emission with the driving voltage was evidenced. Thus, if LECs emit at ca 710 nm when driven at 2.5 V, an emission blue-shifting to 610 nm was obtained upon operating LECs at 7 V, demonstrating a saturation effect resulting in the emission of the host materials [163]. When driven at 3 V, a maximum EQE of 0.75% and maximum luminance of 220 cd/m² were obtained. Examination of the device stability over time revealed LECs to retain 90% of the maximum EQE after four hours of operation (See Table 8).

### 2.6. Phthalocyanines

Phthalocyanines are an important class of metal complexes characterized by strong insolubility in most of the common organic solvents. Phthalocyanine is a fully planar macrocycle comprising 18 aromatic electrons. Due to the planarity of its structure, a strong π-π stacking occurs in the solid state, impeding to disrupt the intermolecular interaction and impeding the dissolution of the complex.

**Figure 13:**
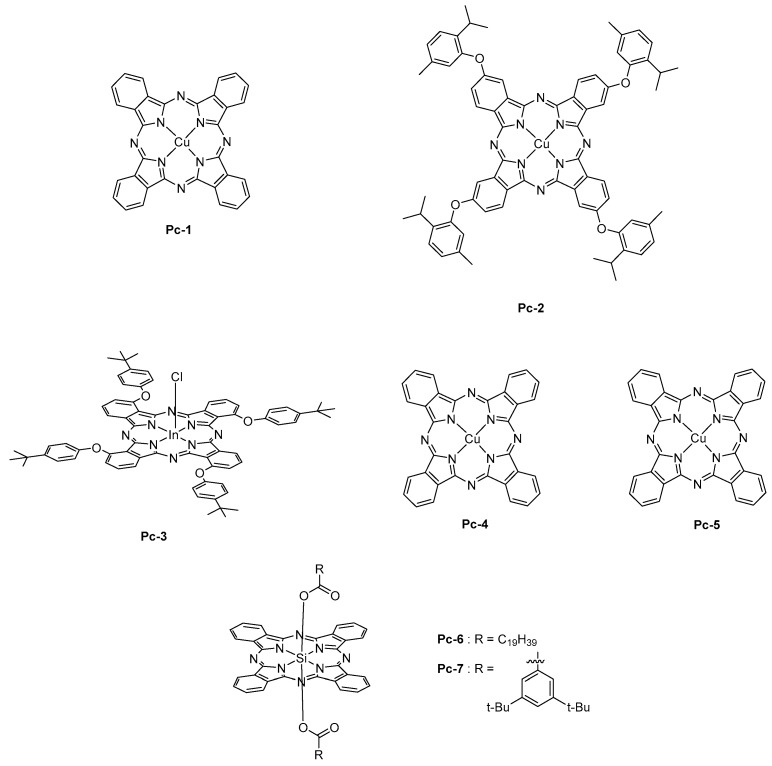
Phthalocyanine-based near-infrared (NIR) emitters for light-emitting diodes.

Face to these considerations, the only way to fabricate OLEDs with phthalocyanines is, thus, the thermal evaporation. As the main advantage, phthalocyanines are extremely stable, even at high temperature so that the thermal deposition was envisioned to construct OLEDs. From the photophysical point of view, a strong absorption band named Q-band is observed around 700 nm [164]. Phthalocyanines also possess the good hole-transport ability, and thus several phthalocyanines have been used as hole-transport materials for OLEDs [165]. The first report mentioning the use of a phthalocyanine as NIR emitter was published in 2006 [166]. In this pioneering work, a copper phthalocyanine (**Pc-1**) doped at 12 wt % into CBP was used, and an EL emission centered at 1100 nm was observed (See Figure 13). Examination of the EL process revealed the excitation of phthalocyanine by direct trapping of electrons and holes. Direct charge trapping by the phthalocyanine was also demonstrated with **Pc-2** [167].

To enhance the EL efficiency, the sensitization of **Pc-1** by an iridium complex, that is, Ir(piq)_2_(acac), enabled to reach a 15-fold enhancement of the EL intensity (see Figure 13) [168]. To get this result, Ir(piq)_2_(acac) was selected as the sensitizer due to the overlap of its emission spectrum with the absorption spectrum of **Pc-1**, its high PLQY, and its long-living excited state lifetime of 1.29 μs. By getting a deeper insight into the EL mechanism, it was found that the excited state lifetime of the Ir complex was shortened for the CBP:Ir(piq)_2_acac: **Pc-1** blended film compared to that of the CBP:Ir(piq)_2_acac film. It could be concluded that the primary mechanism involved in the EL process was an energy transfer from the Ir(piq)_2_(acac) to **Pc-1**. By elaborating devices with a double emissive layer with Ir(piq)_2_(acac) and **Pc-1** into two different layers, almost no improvement of the EL efficiency was detected. Therefore, it was concluded that the proximity of the sensitizer and the emitter was favoring an energy transfer by Dexter mechanism. Recently, the strategy of sensitization of **Pc-1** by triplet harvesting was extended to a purely organic molecule PXZ-TRZ, exhibiting the specific property of thermally activated delayed fluorescence (TADF) [169]. Here again, an energy transfer from the triplet state of this molecule was clearly evidenced. Concerning phthalocyanine, the metal cation introduced in the macrocycle can drastically impact the emission wavelength. Thus, chloroindium phthalocyanine **Pc-3** was found to emit at 880 nm [170], palladium (**Pc-4**) and platinum (**Pc-5**) phthalocyanines at 1025 and 966 nm, respectively [171], whereas an emission around 700 nm was determined for silicon phthalocyanines (**Pc-6** and **Pc-7**) [172].

## 3. Conclusions—Outlook

Since the first reports in the 90′s examining the infrared emission of OLEDs, six main families of metal complexes have been reported in the literature. At present, performances of these devices remain still limited, attributable to the use of non-adapted device structures and charge transport materials. The dramatic difference of performances found for the same emitter while modifying the device structures is the reflection of the difficulty to find the right device architecture and the adapted materials. Over the years, a great deal of efforts has been devoted to developing solution-processed OLEDs due to the high molecular weight of these complexes, which are non-adapted to design vacuum-processed OLEDs. Besides, at present, the most performant devices are still vacuum-processed OLEDs, but the insufficient thermal stability of most of the complexes reported in this review constitutes a major impediment to elaborate highly emissive infrared devices by this process. The preparation of neutral complexes is not always possible as exemplified with ruthenium complexes, and LECs have thus been designed with these non-sublimable complexes. A rapid survey of the results reported in this review reveals platinum complexes to be abandoned for the design of NIR OLEDs. This is certainly attributable to the high cost and the rarity of this transition metal. Most of the references mentioned in this review are pretty old (>10 years ago). Conversely, references concerning iridium complexes are more recent, and the number of NIR iridium complexes (27 mentioned in this review) attests of the interest of the community for this metal. Interest for iridium complexes is notably sustained by the remarkable performances obtained with visible LEDs. The easier color tunability is another parameter to consider. Ruthenium is also known as a revival of interest as numerous works on NIR devices have recently been reported in 2019 with this transition metal. The number of NIR ruthenium complexes (20 complexes) reported in the literature is comparable to that of iridium complexes, attesting of the interest for this metal. Clearly, lanthanides (Er, Nd, Yb, Tm) are not examined anymore for the design of NIR emitters for obvious cost and toxicity issues. Face to the insufficient thermal stability of complexes, such as ruthenium complexes to be vacuum-processed, the low EQE obtained with iridium complexes which seems to be more and more popular for the design of NIR emitters, the search for new structures with more adapted energy levels, higher thermal stability and photoluminescence quantum yield are still actively researched. There is still room for improvements.

## Figures and Tables

**Table 1:** Summary of electroluminescent properties of organic light-emitting diodes (OLEDs) fabricated with Pt-complexes.

Emitters	Device Structure	V_ON_ (V)	Max η_c_^1^ (cd·A^−1^)	Max η_p_^2^ (lm·W^−1^)	CIE Coord. ^3^	Max Brightness (mW·cm^−^²)	EQE (%)	λ_EL_ (nm)	Ref.
**Pt-1**	ITO/NPB (40 nm)/CBP: **Pt-1**(10 wt %, 40 nm)/BCP (40 nm)/Alq_3_ (40 nm)/Mg:Ag (1:10, 100 nm)			0.16 at 2.6 mA/cm²	0.71, 0.28			690, 730, 820	[40]
**Pt-2**	ITO/TPD (70 nm)/CBP (20 nm)/EML (60 nm)/OXA (30 nm)/Ca						10.5	720	[40]
**Pt-3**	ITO/TPD (70 nm)/CBP (20 nm)/EML (60 nm)/OXA (30 nm)/Ca						10.5	715	[40]
**Pt-4**	ITO/TPD (70 nm)/CBP (20 nm)/EML (60 nm)/OXA (30 nm)/Ca		5.5				8.5	705	[40]
**Pt-2**	ITO/75 wt % TPD: 25 wt % PC (60 nm)/CBP (10 nm)/**Pt-2** (30 nm)/OXA (30 nm)/PbO_2_/Ca				0.67, 0.33		14.5	700	[24]
**Pt-5**	ITO/NPB (40 nm)/CBP: **Pt-5** (30 nm, 3 wt %)/BCP (10 nm)/Alq_3_ (30 nm)/LiF (0.5 nm)/Al	3.2	0.32		0.70, 0.29		1.7		[43]
**Pt-6**	ITO/NPB (40 nm)/CBP: **Pt-6** (30 nm, 4.5 wt %)/BCP (10 nm)/Alq_3_ (30 nm)/LiF (0.5 nm)/Al	3.5	0.12		0.70, 0.28		1.3		[43]
**Pt-7**	ITO/NPB (40 nm)/CBP: **Pt-7** (30 nm, 5 wt %)/BCP (10 nm)/Alq_3_ (30 nm)/LiF (0.5 nm)/Al	3.7	0.058		0.71, 0.28		0.95		[43]
**Pt-8**	ITO/HATCN (10 nm)/NPB (50 nm)/mCP (15 nm)/**Pt-8** (20 nm)/TPBi (60 nm)/Liq (2 nm)/Al						24	740	[21]
**Pt-9**	ITO/HATCN (10 nm)/NPB (50 nm)/mCP (15 nm)/**Pt-9** (20 nm)/TPBi (60 nm)/Liq (2 nm)/Al						21	683	[21]
**Pt-10**	ITO/HATCN (10 nm)/NPB (50 nm)/mCP (15 nm)/**Pt-10** (20 nm)/TPBi (60 nm)/Liq (2 nm)/Al						24	669	[21]
**Pt-12**	ITO/NPD (40 nm)/Alq_3_ (40 nm)/Alq_3_: **Pt-12** (6 wt %, 50 nm)/LiF (1 nm)/Al	2.0					6.3	769	[56]
**Pt-12**	ITO/NPD (40 nm)/Alq_3_: **Pt-12** (4 wt %, 25 nm)/BCP (45 nm)/LiF (0.8 nm)/Al						8.5	772	[58]
**Pt-12**	ITO/PEDOT:PSS (40 nm)/PVK:OXD-7: **Pt-12** (100:80:1, 100 nm)/CsF (3 nm)/Al/Ag					0.2		770	[60]
**Pt-12**	ITO/PEDOT:PSS (40 nm)/PVK:PBD: **Pt-12**(6:4, 2 wt %, 110 nm)/LiF(1 nm)/Ca(10 nm)/Al	13.0				1.63	2.07	771	[23]
**Pt-14**	ITO/PEDOT:PSS (40 nm)/PVK:PBD: **Pt-14**(6:4, 2 wt %, 110 nm)/LiF(1 nm)/Ca(10 nm)/Al	17.0				0.67	0.75	898	[23]
**Pt-15**	ITO/PEDOT:PSS (40 nm)/PVK:PBD: **Pt-15**(6:4, 2 wt %, 110 nm)/LiF(1 nm)/Ca(10 nm)/Al	8.3				0.23	0.12	1005	[23]
**Pt-12**	ITO/NPB (40 nm)/Alq_3_: **Pt-12** (4 wt %, 25 nm)/BPhen(80 nm)/LiF(1 nm)/Al	2.2					8.0	773	[23]
**Pt-14**	ITO/NPB (40 nm)/CBP: **Pt-14** (8 wt %, 20 nm)/BPhen(80 nm)/LiF(1 nm)/Al	2.2					3.8	900	[23]
**Pt-16**	ITO/PEDOT:PSS(40 nm)/PVK:PBD:**Pt-16**(6:4, 2 wt %, 110 nm)/LiF(1 nm)/Ca(10 nm)/Al	14.3				1.93	2.49	764	[23]
**Pt-21**	ITO/PEDOT:PSS(40 nm)/PVK:PBD: **Pt-21** (6:4, 2 wt %, 110 nm)/LiF(1 nm)/Ca(10 nm)/Al	14.8				2.19	2.56	774	[23]
**Pt-12**	ITO/PEDOT:PSS(40 nm)/PVK:PBD: **Pt-12** (6:4, 2 wt %, 110 nm)/LiF(1 nm)/Ca(10 nm)/Al					-	-	-	[23]
**Pt-17**	ITO/PEDOT:PSS(40 nm)/PVK:PBD: **Pt-17** (6:4, 2 wt %, 110 nm)/LiF(1 nm)/Ca(10 nm)/Al	13.8				2.23	3.02	774	[23]
**Pt-19**	ITO/PEDOT:PSS(40 nm)/PVK:PBD: **Pt-19** (6:4, 2 wt %, 110 nm)/LiF(1 nm)/Ca(10 nm)/Al	15.6				1.88	2.49	775	[23]
**Pt-18**	ITO/PEDOT:PSS(40 nm)/PVK:PBD: **Pt-18** (6:4, 2 wt %, 110 nm)/LiF(1 nm)/Ca(10 nm)/Al	12.3				2.20	2.70	790	[23]
**Pt-16**	ITO/NPB(40 nm)/Alq_3_:**Pt-16** (4 wt %, 25 nm)/BPhen(80 nm)/LiF(1 nm)/Al	2.3				4.4	9.2	773	[23]
**Pt-21**	ITO/NPB(40 nm)/Alq_3_:**Pt-21** (4 wt %, 25 nm)/BPhen(80 nm)/LiF(1 nm)/Al					-	-	-	[23]
**Pt-20**	ITO/NPB(40 nm)/Alq_3_:**Pt-20** (4 wt %, 25 nm)/BPhen(80 nm)/LiF(1 nm)/Al	2.5				2.1	5.0	777	[23]
**Pt-18**	ITO/NPB(40 nm)/Alq_3_:**Pt-18** (4 wt %, 25 nm)/BPhen(80 nm)/LiF(1 nm)/Al	2.2				3.0	7.8	777	[23]
**Pt-19**	ITO/NPB(40 nm)/Alq_3_:**Pt-19** (4 wt %, 25 nm)/BPhen(80 nm)/LiF(1 nm)/Al	2.5				1.9	3.2	777	[23]
**Pt-18**	ITO/NPB(40 nm)/Alq_3_:**Pt-18** (4 wt %, 25 nm)/BPhen(80 nm)/LiF(1 nm)/Al	2.2				3.2	6.8	792	[23]
**Pt-14**	ITO/PEDOT:PSS(40 nm)/PVK:PBD:**Pt-14**(6:4, 4 wt %, 110 nm)/LiF(1 nm)/Ca(10 nm)/Al	6.0					0.4	896	[69]
**Pt-14**	ITO/NPB(40 nm)/CBP:**Pt-14** (8 wt %, 20 nm)/BPhen(100 nm)/LiF(1 nm)/Al	2.0					3.8	900	[69]
**Pt-24**	ITO/PEDOT:PSS/NPD (30 nm)/TAPC (10 nm)/Alq_3_:4% **Pt-24** (25 nm)/BCP (40 nm)/LiF/Al						1.5	846	[70]
**Pt-23**	ITO/PEDOT:PSS/NPD (30 nm)/TAPC (10 nm)/Alq3:4% Pt-23 (25 nm)/BCP (40 nm)/LiF/Al						2.8	848	[79]

^1^ Maximum current efficiency. ^2^ Maximum power efficiency. ^3^ Chromaticity coordinates. PEDOT:PSS: Poly(3,4-ethylenedioxythiophene)-poly(styrenesulfonate), TPBi: 2,2’,2″-(1,3,5-benzinetriyl)-*tris*(1-phenyl-1-*H*-benzimidazole), PVK: poly(*N*-vinylcarbazole), OXD7: 1,3-*bis*[2-(4-*tert*-butylphenyl)-1,3,4-oxadiazo-5-yl]benzene, BCP: bathocuproine, CBP: 4,4′-bis(*N*-carbazolyl)-1,1′-biphenyl, PBD: 2-(4-*tert*-butylphenyl)-5-(4-biphenylyl)-1,3,4-oxadiazole, TFB: poly[(9,9-dioctylfluorenyl-2,7-diyl)-co-(4,4′-(*N*-(4-*sec*-butylphenyl)diphenylamine)], TmPyPB: 1,3,5-*tris*(3-pyridyl-3-phenyl)benzene, NPB: *N,N*′-di(1-naphthyl)-*N,N*′-diphenyl-(1,1′-biphenyl)-4,4′-diamine, Bphen: bathophenanthroline, DIC-TRZ: 2,4-(diphenyl-6-*bis*(12-phenylindolo)[2,3-*a*]carbazol-11-yl)-1,3,5-triazine, TAPC: 1,1-*bis*[(di-4-tolylamino)phenyl]-cyclohexane, OXA: oxadiazole, Alq3 : tris-(8-hydroxyquinoline)aluminum liq; 8-Hydroxyquinolinolato-lithium.

**Table 2:** Summary of electroluminescent properties of organic light-emitting diodes (OLEDs) fabricated with Ir-complexes.

Emitters	Device Structure	V_ON_ (V)	Radiance (mW·cm^−2^)	EQE (%)	λ_EL_ (nm)	Ref.
**Ir-1**	ITO/PEDOT:PSS(50 nm)/PVK (65%):OXD7(30%):**Ir-1** (5%) (180 nm)/Ba (7 nm)/Al	13		3.07	714	[79]
**Ir-2**	ITO/PEDOT:PSS(50 nm)/PVK (65%):OXD7(30%):**Ir-2** (5%) (180 nm)/Ba (7 nm)/Al	15		1.28	709	[79]
**Ir-3**	ITO/PEDOT:PSS(50 nm)/PVK (65%):OXD7(30%):**Ir-3** (5%) (180 nm)/Ba (7 nm)/Al	14		2.44	714	[79]
**Ir-4**	ITO/PEDOT:PSS (50 nm)/PVK:PBD:**Ir-4** (60 nm)/BCP (20 nm)/Alq_3_ (20 nm)/LiF (1 nm)/Al			0.26	720	[31]
**Ir-5**	ITO/PEDOT:PSS (40 nm)/PVK:30 wt % OXD-7:4 wt % **Ir-5** (60 nm)/TPBi (30 nm)/Ba (4 nm)/Al		54.3 μW/cm²	0.56	697 nm (main peak) 764 nm (sh)	[81]
**Ir-6**	ITO/PEDOT:PSS (30 nm)/TFB (10 nm)/CBP:PBD:**Ir-6** (60:30:10, 40 nm)/TmPyPB (40 nm)/Liq (1 nm)/Al	7.0		1.29	698 nm (main peak) 762 nm (sh)	[82]
**Ir-9**	ITO/PEDOT:PSS (40 nm)/PVK: 40 wt % PBD: 1 wt % **Ir-9** (80 nm)/CsF (1.5 nm)/Al	8.0	444 μW/cm²	3.4	704	[20]
**Ir-7**	ITO/NPB (40 nm)/**Ir-7**:Ga_2_(saph)_2_q_2_ (10–20 wt % 40 nm)/Bphen (30–60 nm)/Mg:Ag		1.8	2.2	777 nm (main peak) 850 (sh)	[83]
**Ir-8**	ITO/NPB (40 nm)/DIC-TRZ:12 wt % **Ir-8** (20 nm)/TPBi (30 nm)/Mg:Ag			4.5	760 nm	[84]
**Ir-10**	ITO/PEDOT:PSS (40 nm)/PVK:PBD (30 wt %): **Ir-10** (20 wt %) (90 nm)/TPBi (30 nm)/Cs_2_CO_3_ (2.3 nm)/Al			0.5	715, 788	[85]
**Ir-11**	ITO/PEDOT:PSS (40 nm)/PVK:PBD (30 wt %): **Ir-11** (20 wt %) (90 nm)/TPBi (30 nm)/Cs_2_CO_3_ (2.3 nm)/Al			0.34	791	[85]
**Ir-12**	ITO/PEDOT:PSS (40 nm)/PVK:PBD (30 wt %: **Ir-12** (20 wt %) (90 nm)/TPBi (30 nm)/Cs_2_CO_3_ (4 nm)/Al			0.30	753	[86]

PEDOT:PSS: Poly(3,4-ethylenedioxythiophene)-poly(styrenesulfonate), TPBi: 2,2’,2″-(1,3,5-benzinetriyl)-*tris*(1-phenyl-1-*H*-benzimidazole), PVK: poly(*N*-vinylcarbazole), OXD7: 1,3-*bis*[2-(4-*tert*-butylphenyl)-1,3,4-oxadiazo-5-yl]benzene, BCP: bathocuproine, CBP: 4,4′-bis(*N*-carbazolyl)-1,1′-biphenyl, PBD: 2-(4-*tert*-butylphenyl)-5-(4-biphenylyl)-1,3,4-oxadiazole, TFB: poly[(9,9-dioctylfluorenyl-2,7-diyl)-co-(4,4′-(*N*-(4-*sec*-butylphenyl)diphenylamine)], TmPyPB: 1,3,5-*tris*(3-pyridyl-3-phenyl)benzene, NPB: *N,N*′-di(1-naphthyl)-*N,N*′-diphenyl-(1,1′-biphenyl)-4,4′-diamine, Bphen: bathophenanthroline, DIC-TRZ: 2,4-(diphenyl-6-*bis*(12-phenylindolo)[2,3-*a*]carbazol-11-yl)-1,3,5-triazine.

**Table 3:** Summary of electroluminescent properties of light-emitting electrochemical cells (LECs) and organic light-emitting diodes (OLEDs) fabricated with Ir-complexes.

Emitters	Device Structure	Current Efficiency (Cd/A)	Brightness (cd/m²)	EQE (%)	CIE coordinates	λ_EL_ (nm)	Ref.
**Ir-13**	ITO/PEDOT:PSS(80 nm)/**Ir-13**:[BMIM^+^:PF_6_^−^] 4:1(100 nm)/Al		18	0.05	0.61, 0.38	650	[88]
**Ir-14**	ITO/PEDOT:PSS(90 nm)/**Ir-14**:[BMIM^+^:PF_6_^−^] 4:1(80 nm)/Al	0.2	19	0.2	-	600	[89]
**Ir-15**	ITO/PEDOT:PSS(90 nm)/**Ir-15**:[BMIM^+^:PF_6_^−^] 4:1(80 nm)/Al	0.2	33	< 0.1	-	600	[89]
**Ir-16**	ITO/PEDOT:PSS(90 nm)/**Ir-16**:[BMIM^+^:PF_6_^−^] 4:1(80 nm)/Al	0.5	50	0.2	-	600	[89]
**Ir-17**	ITO/PEDOT:PSS(90 nm)/**Ir-17**:[BMIM^+^:PF_6_^−^] 4:1(80 nm)/Al	<0.1	low	< 0.1	-	600	[89]
**Ir-18**	ITO/NPB (40 nm)/TCTA:**Ir-18** (20 nm)/TPBi (30 nm)/Mg:Ag (150 nm)/Ag	1.8	5100	0.9	0.52, 0.44	608	[91]
**Ir-19**	ITO/NPB (40 nm)/TCTA:**Ir-19** (20 nm)/TPBi (30 nm)/Mg: Ag (150 nm)/Ag	1.7	2700	0.8	0.55, 0.43	608	[91]
**Ir-20**	ITO/PEDOT:PSS(80 nm)/**Ir-20**:[BMIM^+^:PF_6_^−^] 4:1(80 nm)/Al	-	72	0.13	0.65, 0.34	661	[92]
**Ir-21**	ITO/PEDOT:PSS(80 nm)/**Ir-20**:[BMIM^+^:PF_6_^−^] 4:1(80 nm)/Al	-	471 μW/cm²	0.33	-	705	[92]
**Ir-24**	ITO/PEDOT:PSS (40 nm)/**Ir-24**:[BMIM^+^:PF_6_^−^] 4:1(160 nm)/Ag	-	-	0.036	-	882	[93]
**Ir-27**	ITO/PEDOT:PSS (40 nm)/**Ir-27**:[BMIM^+^:PF_6_^−^] 4:1(160 nm)/Ag	-	-	0.05	-	790	[93]

PEDOT:PSS: Poly(3,4-ethylenedioxythiophene)-poly(styrenesulfonate), BMIM^+^:PF_6_^−^ : 1-Butyl-3-methylimidazolium hexafluorophosphate, TPBi : 2,2’,2″-(1,3,5-benzinetriyl)-*tris*(1-phenyl-1-*H*-benzimidazole).

**Table 4:** Summary of electroluminescent properties of light-emitting electrochemical cells (LECs) and organic light-emitting diodes (OLEDs) fabricated with Ru-complexes.

Emitters	Device Structure	Current Efficiency (Cd/A)	Brightness (mW/Sr m²)	EQE (%)	CIE coordinates	λ_EL_ (nm)	Ref.
**Ru-1**	ITO/**Ru-1** (100 nm)/Au		188	0.31		620	[95]
**Ru-2**	ITO/**Ru-2** (100 nm)/Au		120	0.085		650	[95]
**Ru-3**	ITO/**Ru-3** (100 nm)/Au		49	0.013		780	[95]
**Ru-4**	ITO/**Ru-4** (100 nm)/Au		9	0.075		880	[95]
**Ru-5**	ITO/**Ru-5** (100 nm)/Au		4	0.006		900	[95]
**Ru-6**	ITO/**Ru-6** (100 nm)/Au		5	0.030		945	[95]
**Ru-7**	ITO/**Ru-7** (100 nm)/Au		-			1040	[95]
**Ru-1**	ITO/rGO/[Ru(bpy)_3_].(BF_4_)_2_:Ag		6.89 cd/m²	4.26 × 10^-6^ lm/W		620	[96]
**Ru-8**	ITO/PEDOT:PSS (55 nm)/PVK (60 nm)/**Ru-8** (45 nm)/PBD (30 nm)/Al			0.09	0.587, 0.359	612	[97]
**Ru-9**	ITO/PEDOT:PSS (55 nm)/PVK (60 nm)/**Ru-9** (45 nm)/PBD (30 nm)/Al			0.03	0.550, 0.404	590	[97]
**Ru-10**	ITO/PEDOT:PSS (55 nm)/PVK (60 nm)/**Ru-10** (45 nm)/PBD (30 nm)/Al			0.11	0.630, 0.365	583	[97]
**Ru-11**	ITO/PEDOT:PSS (55 nm)/PVK (60 nm)/**Ru-11** (45 nm)/PBD (30 nm)/Al			0.04	0.630, 0.365	575	[97]
**Ru-12**	ITO/PEDOT:PSS (55 nm)/PVK (60 nm)/**Ru-12** (45 nm)/PBD (30 nm)/Al			0.17	0.559, 0.413	600	[97]
**Ru-13**	ITO/PEDOT:PSS (55 nm)/PVK (60 nm)/**Ru-13** (45 nm)/PBD (30 nm)/Al			0.31	0.531, 0.466	568	[97]
**Ru-14**	ITO/PEDOT:PSS/**Ru-14**:PMMA/Al			0.005			[103]
**Ru-15**	ITO/PEDOT:PSS/**Ru-14**:PMMA/Al			0.005			[103]
**Ru-16**	ITO/**Ru-16** (126 nm)/Ga:In	0.27	1066 cd/m²	1.40	0.730, 0.269	664	[108]
**Ru-17**	ITO/**Ru-17** (126 nm)/Ga:In	0.19	589 cd/m²	0.93	0.734, 0.265	695	[108]
**Ru-18**	ITO/**Ru-18** (126 nm)/Ga:In	0.26	878 cd/m²	1.15	0.722, 0.277	644	[108]
**Ru-19**	ITO/**Ru-19** (126 nm)/Ga:In	0.12	1921 cd/m²	0.141	0.652, 0.315	635	[111]
**Ru-20**	ITO/**Ru-20** (126 nm)/Ga:In	0.34	2224 cd/m²	0.682	0.628, 0.309	690	[111]

PEDOT:PSS: Poly(3,4-ethylenedioxythiophene)-poly(styrenesulfonate), PVK: poly(*N*-vinylcarbazole), PMMA :Poly(methyl methacrylate), PBD: 2-(4-*tert*-butylphenyl)-5-(4-biphenylyl)-1,3,4-oxadiazole, rGO : reduced graphene oxide.

**Table 5:** Summary of electroluminescent properties of organic light-emitting diodes (OLEDs) fabricated with Er-complexes.

Emitters	Device Structure	V_ON_ (V)	Radiance (mW·cm^−2^)	λ_EL_ (nm)	Ref.
**Er-2**	ITO/PVK:**Er-2**/Al:Li/Ag			1540	[127]
**Er-3**	p-Si substrate/SiO_2_ (1.5 nm)/NPB (60 nm)/Bphen: **Er-3** (20 nm)/Bphen (25 nm)/CsPh (25 nm)/Sm (15 nm)/Au			1540	[128]
**Er-5**	ITO/PEDOT:PSS (70 nm)/active layer (170 nm)/Ca/Al	7.0		1535	[129]
**Er-5**	ITO/PEDOT:PSS (70 nm)/active layer (170 nm)/Ca/Al	7.0.		1540	[132]
**Er-4**	ITO/PEDOT:PSS (70 nm)/active layer (97 nm)/Ca/Al	6.5		1540	[132] [130]
**Er-6**	ITO/PEDOT:PSS (100 nm)/**Er-6** (75 nm)/Ca/Al	7.0		1540	[133]
**Er-7**	ITO/PEDOT:PSS (100 nm)/**Er-7** (70 nm)/Ca/Al	4.0		1540	[133]
**Er-8**	ITO/NPB (30 nm)/**Er-8** (40 nm)/TPBi (30 nm)/LiF (0.1 nm)/Al	14.0	0.069	1534	[134]

PEDOT:PSS: Poly(3,4-ethylenedioxythiophene)-poly(styrenesulfonate), TPBi: 2,2’,2″-(1,3,5-benzinetriyl)-*tris*(1-phenyl-1-*H*-benzimidazole), PVK: poly(*N*-vinylcarbazole), NPB: *N,N*′-di(1-naphthyl)-*N,N*′-diphenyl-(1,1′-biphenyl)-4,4′-diamine, CsPh: not defined in the article.

**Table 6:** Summary of electroluminescent properties of organic light-emitting diodes (OLEDs) fabricated with Nd-complexes.

Emitters	Device Structure	V_ON_ (V)	Radiance (mW·cm^−2^)	EQE (%)	λ_EL_ (nm)	Ref.
**Nd-1**	ITO/TPD (50 nm)/**Nd-1** (25 nm)/Alq_3_ (50 nm)/Mg:Ag (10:1)	15.0			890, 1070, 1350	[135]
**Nd-1**	ITO/TPD (40 nm)/**Nd-1** (20 nm)/BCP (40 nm)/Mg:Ag (10:1)				890, 1070, 1350	[136]
**Nd-2**	ITO/TPD (50 nm)/**Nd-2** (60 nm)/Al				900, 1060, 1320	[137]
**Nd-3**	ITO/PEDOT:PSS (70 nm)/PVK: **Nd-3** (100 nm)/Ca (100 nm)/Al (100 nm)		8.5 nW/mm²	0.007	1065	[138]
**Nd-4**	ITO/NPB (50 nm)/CBP: **Nd-4** (7:1, 20 nm)/BCP (35 nm)/LiF (1 nm)/Al		25 μW/cm²	0.019	880, 1064, 1330	[139]
**Ir1-Nd-5**	ITO/NPB (30 nm)/emitting layer (40 nm)/BCP (10 nm)/Alq_3_ (30 nm)/Mg0.9Ag0.1 (200 nm)/Ag		6.1 μW/cm²		1060	[140]

PEDOT:PSS: Poly(3,4-ethylenedioxythiophene)-poly(styrenesulfonate), PVK: poly(*N*-vinylcarbazole), BCP: bathocuproine, CBP: 4,4′-bis(*N*-carbazolyl)-1,1′-biphenyl, Alq3 : *tris*-(8-hydroxyquinoline)aluminum.

**Table 7:** Summary of electroluminescent properties of organic light-emitting diodes (OLEDs) fabricated with Yb-complexes.

Emitters	Device Structure	V_ON_ (V)	Radiance (mW·cm^−2^)	EQE (%)	λ_EL_ (nm)	Ref.
**Yb-1**	ITO/NPB (25 nm)/**Yb-1** (10%): TcTa (40 nm)/BCP (15 nm)/Alq_3_ (10 nm)/LiF (0.5 nm)/Al		19.29 μW/cm²		980	[143]
**Yb-2**	ITO/NPB (20 nm)/**Yb-2** (20 nm)/**Yb-2** (40 nm)/Alq_3_ (20 nm)/LiF/Al		1.47 μW/cm²		980	[145]
**Yb-3**	ITO/Polystyrene: **Yb-3** (33 wt %)/Ca (5 nm)/Al	4.0	0.6 μW/cm²		977	[146]
**Yb-3**	ITO/PEDOT:PSS (20 nm)/PPP-OR11: **Yb-3** (10 wt %, 100 nm)/Ca (5 nm)/Al	4.5		0.04	977	[30]
**Yb-3**	ITO/PEDOT:PSS (40 nm)/PPP-OR11: **Yb-3** (10 wt %, 50 nm)/Ca (5 nm)/Al		10 μW/cm²	0.00013	977	[147]
**Yb-9**	ITO/TPD (40 nm)/**Yb-9**: TPD (1:1, 40 nm)/**Yb-9** (60 nm)/Ag:Mg	4.5			980	[151]
**Yb-4**	ITO/β-NPB (25 nm)/**Yb-4** (10%):TcTa (40 nm)/BCP (15 nm)/Alq_3_ (10 nm)/LiF (0.5 nm)/Al	7.6	22.48 μW/cm²			[149]
**Yb-5**	ITO/β-NPB (25 nm)/**Yb-5** (10%):TcTa (40 nm)/BCP (15 nm)/Alq_3_ (10 nm)/LiF (0.5 nm)/Al	7.7	12.13 μW/cm²			[149]
**Yb-6**	ITO/β-NPB (25 nm)/**Yb-6** (10%):TcTa (40 nm)/BCP (15 nm)/Alq_3_ (10 nm)/LiF (0.5 nm)/Al	7.9	9.60 μW/cm²			[149]
**Yb-7**	ITO/TPD (50 nm)/**Yb-7** (60 nm)/Al				977	[150]
**Yb-10**	ITO/PEDOT:PSS/**Yb-10** (30 nm)/TAZ (10 nm)/Ca/Al	4.0		0.21%	978	[152]
**Yb-11**	ITO/α-NPD (30 nm)/mCP (10 nm)/**Yb-11**:DPEPO (30 nm)/DPEPO (10 nm)/TPBi (40 nm)/LiF (0.8 nm)/Al			0.15%	1000–1100	[153]

PEDOT:PSS: Poly(3,4-ethylenedioxythiophene)-poly(styrenesulfonate), TPBi: 2,2’,2″-(1,3,5-benzinetriyl)-*tris*(1-phenyl-1-*H*-benzimidazole), BCP: bathocuproine, β-NPB: *N,N*′-di(1-naphthyl)-*N,N*′-diphenyl-(1,1′-biphenyl)-4,4′-diamine, Alq3 : *tris*-(8-hydroxyquinoline)aluminum, PPP-OR11: *bis*-alkoxy-substituted poly(*p*-phenylene), TPD: N*,N′-bis*(3-methylphenyl)-*N,N*′-diphenylbenzidine, TcTa: *tris*(4-carbazoyl-9-ylphenyl)amine, DPEPO: *bis*[2-(diphenylphosphino)phenyl]ether oxide.

**Table 8:** Summary of electroluminescent properties of organic light-emitting diodes (OLEDs) fabricated with various complexes.

Emitters	Device Structure	V_ON_ (V)	Radiance (mW·cm^−2^)	EQE (%)	λ_EL_ (nm)	Ref.
**Os-1**	ITO/α-NPD (40 nm)/Alq_3_ :**Os-1** (6 wt %, 25 nm)/TAZ (45 nm)/LiF (0.5 nm)/Al	2.4	65 μW/cm²	1.5	814	[19]
**Os-2**	ITO/α-NPD (40 nm)/Alq_3_: **Os-2** (6 wt %, 25 nm)/TAZ (45 nm)/LiF (0.5 nm)/Al	3.0	93 μW/cm²	2.7	718	[158]
**Os-3**	ITO/**Os-3**:Ru(bpy)_3_^2+^ (100 nm)/Au		220 cd/m²	0.75		[159]
**Pc-1**	ITO/NPB (30 nm)/CBP:**Pc-1** (12 wt %, 30 nm)/BCP (20 nm)/Alq_3_ (20 nm)/Al				1100	[166]
**Pc-1**	ITO/m-MTDATA (20 nm)/TPD (20 nm)/CBP: **Pc-1**:Ir(piq)_2_acac (10, 12 wt %, 30 nm)/TPBI (40 nm)/LiF(1 nm)/Al				1120	[168]
**Pc-3**	ITO/PVK: **Pc-3** (40 nm)/BCP (18 nm)/Alq_3_ (15 nm)/Al				880	[170]
**Pc-6**	ITO/PEDOT:PSS (30 nm)/PVK (30 nm)/CBP:PBD: **Pc-6** [30:(70−x):x; x = 1, 5, or 10 wt %; **Pc-6**; 30 nm]/B3PYMPM (50 nm)/Ca (20 nm)/Al	9.9		0.64	701	[171]
**Pc-4**					1025	[171]
**Pc-5**					966	[171]
**Pc-7**	ITO/PEDOT:PSS (30 nm)/PVK (30 nm)/CBP:PBD: **Pc-7** [30:(70 − x):x; x = 1, 5, or 10 wt %; **Pc-7**; 30 nm]/B3PYMPM (50 nm)/Ca (20 nm)/Al	7.4		1.40	700	[172]

PEDOT:PSS: Poly(3,4-ethylenedioxythiophene)-poly(styrenesulfonate), TAZ: 3-(biphenyl-4-yl)-5-(4-tert-butylphenyl)-4-phenyl-4H-1,2,4-triazole, BCP: bathocuproine, Alq3 : tris-(8-hydroxyquinoline)aluminum liq; 8-Hydroxyquinolinolato-lithium, PBD: 2-(4-*tert*-butylphenyl)-5-(4-biphenylyl)-1,3,4-oxadiazole, B3PYMPM: 4,6-*bis*(3,5-di(pyridin-3-yl)phenyl)-2-methylpyrimidine, CBP: 4,4′-bis(*N*-carbazolyl)-1,1′-biphenyl, TPBi: 2,2′,2″-(1,3,5-benzinetriyl)-tris(1-phenyl-1-*H*-benzimidazole), Ir(piq)_2_acac: *bis*(1-phenylisoquinoline) (acetyl-acetonate)iridium (III).
